# TLR4 Signaling Selectively and Directly Promotes CGRP Release from Vagal Afferents in the Mouse

**DOI:** 10.1523/ENEURO.0254-20.2020

**Published:** 2021-01-15

**Authors:** Lin Jia, Syann Lee, Jessica A. Tierney, Joel K. Elmquist, Michael D. Burton, Laurent Gautron

**Affiliations:** 1 Center for Hypothalamic Research and Department of Internal Medicine, The University of Texas Southwestern Medical Center, Dallas, TX 75390; 2 Neuroimmunology and Behavior Laboratory, Department of Neuroscience, School of Behavioral and Brain Sciences, and Center for Advanced Pain Studies, University of Texas at Dallas, Richardson, TX 75081

**Keywords:** Cre-loxP, innate immunity, neuropeptide, vagus nerve

## Abstract

There has been a long-standing debate regarding the role of peripheral afferents in mediating rapid-onset anorexia among other responses elicited by peripheral inflammatory insults. Thus, the current study assessed the sufficiency of peripheral afferents expressing toll-like receptor 4 (TLR4) to the initiation of the anorexia caused by peripheral bacterial lipopolysaccharide (LPS). We generated a Tlr4 null (Tlr4^LoxTB^) mouse in which Tlr4 expression is globally disrupted by a loxP-flanked transcription blocking (TB) cassette. This novel mouse model allowed us to restore the endogenous TLR4 expression in specific cell types. Using Zp3-Cre and Na_v_1.8-Cre mice, we produced mice that express TLR4 in all cells (Tlr4^LoxTB^ X Zp3-Cre) and in peripheral afferents (Tlr4^LoxTB^ X Na_v_1.8-Cre), respectively. We validated the Tlr4^LoxTB^ mice, which were phenotypically identical to previously reported global TLR4 knock-out mice. Contrary to our expectations, the administration of LPS did not cause rapid-onset anorexia in mice with Na_v_1.8-restricted TLR4. The later result prompted us to identify Tlr4-expressing vagal afferents using *in situ* hybridization (ISH). *In vivo*, we found that Tlr4 mRNA was primarily enriched in vagal Na_v_1.8 afferents located in the jugular ganglion that co-expressed calcitonin gene-related peptide (CGRP). *In vitro*, the application of LPS to cultured Na_v_1.8-restricted TLR4 afferents was sufficient to stimulate the release of CGRP. In summary, we demonstrated using a new mouse model that vagally-expressed TLR4 is selectively involved in stimulating the release of CGRP but not in causing anorexia.

## Visual Abstract

**Figure:**
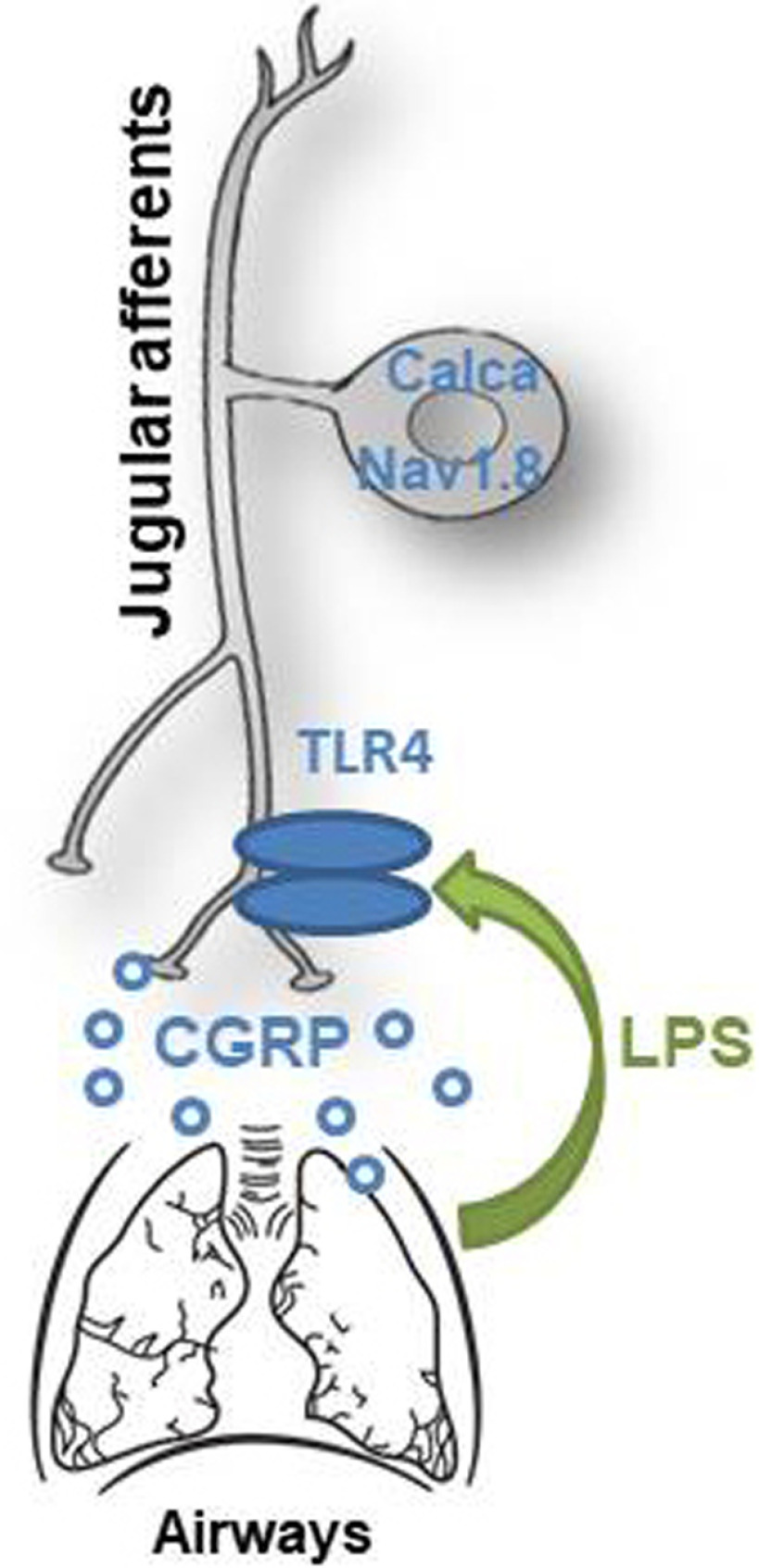

## Significance Statement

Using a new transgenic mouse model, our data establish that toll-like receptor 4 (TLR4) is both sufficient and required for the release of calcitonin gene-related peptide (CGRP) from a subset of vagal afferents. This finding may be relevant to the understanding of how bacterial infections modulate nerves.

## Introduction

Toll-like receptor 4 (TLR4) is the main membrane receptor for lipopolysaccharides (LPS), which are endotoxins derived from the outer membrane of Gram-negative bacteria (Poltorak et al., 1998a,b, 2000; Chow et al., 1999). Hence, the immunologic effects of LPS are blunted in TLR4-deficient mice (Hoshino et al., 1999). That said, other membrane proteins, including the myeloid differentiation factor 2 (Park et al., 2009) and TRP channels (Meseguer et al., 2014; Alpizar et al., 2017) also contribute to LPS signaling. Upon exposure to LPS, anorexia ensues (Kent et al., 1992; Porter et al., 1998a) as the direct result of the actions of proinflammatory molecules on the CNS (Layé et al., 1994; Grossberg et al., 2010; Daniel et al., 2016; Essner et al., 2017). While the anorexic effects of LPS occur within minutes (Liu et al., 2016), LPS does not cross the blood-brain barrier (Banks and Robinson, 2010), suggesting that a peripheral neural mechanism involving vagal afferents may contribute to LPS-induced anorexia. This is because the peripheral endings of vagal afferents are outside the blood-brain barrier and directly accessible to bacterial products located in vagally-innervated tissues. Experiments conducted in deafferented animals are in agreement with this view (Bluthé et al., 1994; Bret-Dibat et al., 1995; Layé et al., 1995; Konsman et al., 2000). However, it was never clear whether LPS could directly act on peripheral neurons until TLR4 was found to be expressed in peripheral afferents, including those in the vagus nerve (Hua et al., 1996; Hosoi et al., 2005; Ochoa-Cortes et al., 2010; de Lartigue et al., 2011; Diogenes et al., 2011; Due et al., 2012; Kunda et al., 2014; Helley et al., 2015; Li et al., 2015; Boonen et al., 2018). Moreover, the ability of isolated sensory neurons to directly and rapidly respond to LPS has been reported in different species using electrophysiology and calcium signaling (Riley et al., 2013; Meseguer et al., 2014). The *in vivo* administration of LPS has also been reported to produce changes in the neuropeptide expression and firing rate of vagal afferents (Huang and Lai, 2003; Liu et al., 2007). Thus, it is plausible that this mechanism allows peripheral sensory neurons to detect infections in a rapid manner (Clatworthy and Grose, 1999; Chiu et al., 2013; Soldano et al., 2016). Finally, it must be noted that within the brain, TLR4 is expressed in endothelial and glial cells, but not in neurons (Laflamme and Rivest, 2001; Chakravarty and Herkenham, 2005; Kigerl et al., 2007). The latter observation suggests that peripheral afferents are likely the only neurons in the body able to sense changing levels of TLR4 activators in a direct manner.

Other findings contradict the view that TLR4 is present and functional in vagal afferents. Another RNA sequencing study of vagal afferents failed to detect any vagal afferents with significant Tlr4 mRNA expression (Wang et al., 2017). This is without mentioning that several laboratories have demonstrated that interrupting vagal afferents does not prevent the host of physiological responses to peripheral LPS, including anorexia (Bret-Dibat et al., 1997; Schwartz et al., 1997; Hansen et al., 2000; Martin et al., 2000; Wieczorek et al., 2005). Considering that vagal afferents are exquisitely sensitive to a wide range of proinflammatory molecules (Ek et al., 1998; Yu et al., 2005; Feldman-Goriachnik et al., 2015), it remains possible that the aforementioned cellular and physiological effects of LPS are not directly mediated by TLR4 signaling in vagal afferents themselves. Given the above uncertainties in the field, the current study aimed to reassess the sufficiency of vagal afferents TLR4 to select LPS-induced responses using a newly generated transgenic mouse model.

## Materials and Methods

### Mice

Mice were all housed in a barrier facility in a light-controlled and temperature-controlled environment [zeitgeber (ZT)0 = 6 A.M.] with *ad libitum* access to standard chow, unless specified otherwise (Harlan Teklad TD.2016 Global). Experimental mice were males, whereas females were only used for breeding purposes. Single housing was required during the course of food intake studies. Otherwise, animals were group housed at all time. All the procedures in this study were approved by our Institutional Animal Care and Use Committees and in accordance with the Society’s Policies on the Use of Animals and Humans in Neuroscience Research.

#### Wild-type (WT) C57Bl/6 mice

A total of six male mice, between six and eight weeks of age, on a pure C57Bl/6 background were used for chromogenic *in situ* hybridization (ISH) experiments. These mice were obtained from our Animal Resource Center.

#### Na_v_1.8-Cre^ChR2-YFP^ reporter mice

We also generated and genotyped Na_v_1.8-Cre-ChR2-YFP mice carrying one Cre allele and one ChR2-YFP allele. A total of four males between six and eight weeks of age, were used for chromogenic ISH combined with GFP immunostaining experiments.

#### Generation of novel Cre-reactivated TLR4-null mice (Tlr4^LoxTB^)

To generate Tlr4^LoxTB^ mice, BAC clones containing the murine Tlr4 gene derived from the 129/Sv strain were obtained from the Sanger Institute. The BAC DNA clone number bMQ58F22 was electroporated into EL350 bacteria. First, the loxP sequences present in the BAC backbone were removed by homologous recombination as previously described (Lee et al., 2001). Then the validated loxP-flanked transcription blocking (TB) sequences (Berglund et al., 2012) were amplified by PCR using primers 5′-GCCTCATTTAGAAGTGGAATGATAGAAACTCACAGAAATTAATGGGTTCCCAAGATCATGGGTACCCGCGCCTAGTCGACTTCGAATAACTT-3′ and 5′-TGTTAAAAAGAATATCGCAAGAGGAATCCATGGAGCCATGTTAACTCTCCATTCTTCCTGGGTGGCGGCCGCTTAGTTTA-3′. The amplicon was then inserted between exons 2 and 3 of the Tlr4 gene by homologous recombination in EL350 cells (Lee et al., 2001). Kanamycin-resistant clones were selected and recombination was verified by PCR using the following primers: 5′-CTGGACAAACAGTGGCTGGA-3′ and 5′-GTCATAGATGCATGCCAGATACA-3′. Next, the Amp resistance sequence in the EL350 clones with correctly targeted Tlr4 BAC DNA (LoxTB-Tlr4) was replaced with a Zeocin resistance cassette. pZErO−1 vector (Invitrogen) was used to amplify the Zeocin resistance sequence by PCR, and the amplicon was inserted by homologous recombination. Zeocin resistant clones were selected and recombination was verified by PCR. Following this, the pGEM-T easy vector (Promega) was used as a template to generate an amplicon containing two Tlr4 homology arms using primers: 5′-CCATTCAAACTGGAACATAGCCACCTAATTATTTGTCTCTTGTTAGCCAAGTGAAATAGCgcggccgcCCAACGCGTTGGATGCATAGC-3′ and 5′-GTGTGGGTGAAGGTAAGAGTAGCTGTATGCATTACATAGATGTATGAAATTGTCAAAGAGCGGTATTTTCTCCTTACGCATC-3′. The Tlr4^LoxTB^ gene was inserted into the pGEM-T easy vector through homologous recombination and positive clones were verified by PCR using primers: 5′-ACCTGATGCGGTGTGAAATAC-3′ and 5′-AGGAAACAGCTATGACCATGA-3′. Sequencing of the amplicons was performed and enzymatic digestions were conducted to confirm on-target homologous recombination, the sequences of the loxP-flanked TB cassette, and Tlr4 native sequences. The targeting vector, which consisted of the LoxTB-Tlr4 gene flanked by 3.2-kb (left) and 5.2-kb (right) Tlr4 homology arms was prepared using a commercially available kit (QIAGEN), linearized by PvuI enzymatic digestion and electroporated into 129/SvJ ES cells by the transgenic core facility at The University of Texas Southwestern Medical Center. To identify the recombinant clones, genomic DNA was extracted from ES cells as previously described (Berglund et al., 2012) and used for long PCR assays to discriminate between ES clones hosting WT and loxP-modified Tlr4 allele. Correct targeting was further confirmed by a custom multiplex TaqMan quantitative PCR (qPCR) assay. Briefly, extracted DNA was used as template (5–100 ng). Melanocortin 4 receptor (MC4R) was used as an endogenous reference (ID Mm00457483_s1, FAM dye-labeled probe; Applied Biosystems). Custom primers (5′-TCCTAACAGAAAGTGGAAACTTGAG-3′ and 5′-AGGAATCCATGGAGCCATGTTA-3′) and VIC dye-labeled probe (5′-CCCAAGATCATGCAGGAAGAAT-3′) from Biosearch Technologies were multiplexed with the MC4R assay. DNA-based qPCRs were conducted on an Applied Biosystems PRISM 7900HT sequence detection system. Correctly targeted recombinant ES cells were injected into blastocysts of C57Bl/6 mice. Chimeric male mice (F0) were crossed to C57Bl/6 female mice and their pups (F1) were screened for germ line transmission by PCR using the same strategy as for genotyping. F1 pups bearing the Cre-reactivatable Tlr4-null allele (Tlr4^LoxTB^) were then crossed with zona pellucida 3 (Zp3)-Cre transgenic mice (stock #003651 C57BL/6-Tg(Zp3-cre)93Knw/J) to restore endogenous TLR4 expression in all tissues (Tlr4^LoxTB^ X Zp3-Cre). Mice carrying a WT and/or Cre-mediated recombination allele (Tlr4^LoxTB^ X Zp3-Cre) were genotyped by primer pairs P1 (5′-CTGACTGGTGTGAAGTGGAATATC-3′) and P3 (5′- GTCATAGATGCATGCCAGATACA-3′). Primers P2 (5′-CTGGACAAACAGTGGCTGGA-3′) and P3 were used to screen the Tlr4^LoxTB^ allele. Globally reactivated mice were used for validation studies and feeding tests. The group size is indicated in figure legends.

#### Tlr4^LoxTB^ X Na_v_1.8-Cre mice

F1 pups bearing the Cre-reactivated Tlr4^LoxTB^ allele were then crossed with Na_v_1.8-Cre mice to produce mice with endogenous TLR4 reactivation only in Na_v_1.8 neurons (Tlr4^LoxTB^ X Na_v_1.8-Cre). These mice are referred to as Na_v_1.8-restricted Tlr4 mice. For all studies, breeding was set up to obtain the following groups: control, Tlr4^LoxTB^, and Na_v_1.8-restricted Tlr4 mice. Controls consisted of mice expressing one copy of the Na_v_1.8-Cre allele. Tlr4^LoxTB^ mice expressed two copies of the Tlr4^LoxTB^ allele without Cre. Na_v_1.8-restricted Tlr4 mice expressed two copies of the Tlr4^LoxTB^ allele and one Cre allele. To validate our ISH probe, we also compared the ganglia from two Tlr4^LoxTB^ mice to that of two globally reactivated mice (Tlr4^LoxTB^ x Zp3-Cre). Mice were maintained on a mixed (C57Bl/6 and 129) genetic background. Male mice entered the experiments at 8–16 weeks of age and were used for feeding and calcitonin gene-related peptide (CGRP) studies. The group size is indicated in figure legends.

### LPS preparation and dosage

For animal and culture experiments, we kept 1 mg/ml aliquots of LPS solution (Sigma L2880 *Escherichia coli* 055:B5) in sterile pyrogen-free 0.9% saline (Sigma) at −20°C. When needed, LPS was diluted 100 times in sterile saline for animal studies or in sterile media for culture studies. In all our feeding studies, LPS was administered at a single dose of 100 μg/kg of body weight (intraperitoneal). This moderate dose is sufficient to induce rapid and transient anorexia in the mouse (Bret-Dibat and Dantzer, 2000; Lawrence et al., 2012; Liu et al., 2016). A dose of 2 mg/kg (intraperitoneal) was used to stimulate Nfkbia expression in the nodose ganglion. This dosage was chosen based on a prior study demonstrating LPS-induced gene expression changes in the nodose ganglion (Huang and Lai, 2003). A dose of 500 ng/ml of LPS was used for nodose organotypic culture studies (Marrone et al., 2017).

### LPS-induced inflammatory response in mice

WT, Tlr4^LoxTB^, and Tlr4^LoxTB^ X Zp3-Cre mice (7–10 weeks old) were treated with LPS (1 or 0.5 mg/kg body weight) by intraperitoneal injection. Blood was collected 1.5 h after injection by tail bleeding. Plasma TNFα concentrations were determined using the MILLIPLEX MAP Mouse Cytokine/Chemokine panel (Millipore).

### Genomic DNA isolation

Genomic DNA was isolated from tails of six-week-old mice using REDExtract-N-Amp Tissue PCR kit (Sigma) following the manufacturer’s instructions.

### Feeding studies

Individually-housed control, Tlr4 deficient, and reactivated mice were fasted overnight. At ∼9 A.M., fasted mice were weighed and immediately treated with either LPS or sterile saline. At the same time, mice were refed with chow. An experimenter continuously recorded the feeding behavior of each mouse over 60 min following treatment. At the end of the 60-min period, food pellets and body weight were measured. Data were used to calculate the total amount of time spent eating in seconds.

### LPS-induced gene expression studies by qPCR

Mice were fasted overnight. The next morning, body weights were measured to determine the injection volume of saline or LPS. Then, saline or LPS was administered at the single dosage of 2 mg/kg, intraperitoneally. One hour postinjection, mice received an overdose of chloral hydrate (500 mg/kg, i.p.) and their nodose-jugular ganglia were rapidly removed and frozen in liquid nitrogen. Total RNAs were extracted using RNA Stat60 (Teltest) according to the manufacturer’s instructions. RNA concentration and quality were determined by NanoDrop 1000 Spectrophotometer (Thermo Scientific). Complementary DNA was synthesized using the High Capacity cDNA kit and was performed at 25°C for 10 min, 37°C for 120 min, and 85°C for 5 min (Applied Biosystems). Primers for Tlr4 (ID: Mm0445274_m1), Nfkbia (ID: Mm00477798_m1), and 18s (ID: Hs99999901_s1) were purchased from Applied Biosystems. The mRNA contents were normalized to 18s mRNA levels. qPCR was performed in an ABI Prism 7900HT sequence detection system (Applied Biosystems) using TaqMan Master Mix (Applied Biosystems) and 10 ng of synthesized cDNA. Samples were run in triplicates. The relative amounts of all mRNAs were calculated using the ΔΔCT assay.

### ISH, microscopy, and digital images analysis

Mice received an overdose of chloral hydrate (500 mg/kg, i.p.), before being transcardiacally perfused with 10% formalin (Sigma). The nodose-jugular ganglionic mass was rapidly dissected and kept in formalin for an additional 24 h at 4°C. Fixed ganglia were next incubated overnight in a solution of 20% sucrose in PBS (pH 7.4) at 4°C before being frozen on a bed of dry ice. Series of 14-μm sections were collected on SuperFrost slides using a cryostat. Tissue was stored at −80°C for less than a couple of weeks. The tissue was processed for ISH following the manufacturer’s instructions (Advanced Cell Diagnostics) with only minor adjustments to the pretreatment. Specifically, slides were baked for ∼30 min at 60°C before starting the pretreatment. In addition, the target retrieval solution was heated up to 87°C instead of 99°C. Table 1 summarizes the probes, chromogenic labels, and reagents used in the current study. Tissue sections labeled for Tlr4 alone or Tlr4 combined with Calca (CGRP precursor gene) were counterstained with a hematoxylin solution (GHS132 Sigma). Tissue sections from Na_v_1.8-Cre^ChR2-YFP^ mice were not counterstained, but further processed for GFP immunohistochemistry. Briefly, after several PBS washes, the sections were incubated overnight at room temperature in a chicken anti-GFP primary antiserum (Aves Laboratory; GFP-10 120; RRID:AB_10000240; 1:1000) in 3% normal donkey serum with 0.25% Triton X-100 in PBS. On the following day, sections were washed and incubated for 1 h in a biotinylated anti-chicken secondary antibody (catalog #703065155; Jackson ImmunoResearch; 1:1000), followed by Streptavidin Alexa Fluor 488 (Invitrogen, #S11223; 1:1000). All our slides were coverslipped with EcoMount mounting medium (Biocare Medical, LLC EM897L).

**Table 1:** List of reagents used for ISH

ISH RNAscope reagents from ACD						
Gene style name(s)	Accession number	Probe region	Catalog number channel	Chromogenic labels	Pretreatment	Kits
Tlr4-C1	NM_021297.2	3006–3775	316801-C1	Fast-Red	RNAscope Hydrogen Peroxide RNAscope Target Retrieval RNAscope Protease Plus	RNAscope 2.5 HD Reagent Kit-RED assay
Calca-C1	NM_007587.2	44–995	417961-C1	HRP-based Green	RNAscope Hydrogen Peroxide RNAscope Target Retrieval RNAscope Protease III	RNAscope2.0 2-plex
Tlr4-C2	NM_021297.2	2404–3775	316801-C2	AP-based Fast Red		

Bright-field images were captured using a Zeiss microscope (Imager ZI) attached to a digital camera (Axiocam). A camera lucida attached to the microscope was also used to draw tissue sections. Drawings were digitalized and exported to Adobe Illustrator Artwork 15.1. The Zeiss microscope (Imager ZI) was also used for manual counts of the ISH signals for cell profiles positive for Tlr4/H&E as well as Tlr4/Calca. Cell profiles were counted on digital images by an experimenter unaware of our experimental design. Fluorescent digital images were all acquired with the 20× objective of a Zeiss microscope (Imager ZI) or the 63× objective of a Leica TCS SP5 confocal microscope (The University of Texas Southwestern Live Cell Imaging Core). Scanning parameters were adjusted appropriately to improve the signal/background. We collected Z-stacks separated by ∼0.35–0.45 μm in a 512 × 512-pixel format. NIH ImageJ software was used to generate our final TIFF images with combined Z stacks. Adobe Photoshop CS2 software was used to combine digital images into plates with annotations. The contrast and brightness of digital images were uniformly adjusted. Neuronal versus non-neuronal profiles were usually easily identifiable by the size, shape and intensity of the counterstaining of their nuclei. Our counts should be considered estimates rather than absolute counts. Cells that were considered doubly labeled included profiles with accumulation of Tlr4 mRNA (at least one red dot per profile) within the boundaries of one Calca-positive profile. In addition, the circumference of each identified cell profile was assessed using the measurement tools in ImageJ. Images acquired by confocal microscopy were further evaluated for doubly labeled cells using plot of fluorescence intensities. Briefly, we manually traced a line across identified cell profiles using the ImageJ software (NIH, Fiji version). Plot profiles of gray values were generated for each color channel (overlapping red and green). Representative plot profiles were included in our figures. Cells with colocalized fluorescence displayed overlapping red and green plots of fluorescence across the line profile. Cells without staining or minimal colocalization displayed a flat plot profile in at least one channel. Colocalization data were expressed as the mean percentage of identified cell profiles ± SEM, and the absolute numbers of counted profiles were included in the figures.

### Organotypic nodose ganglion and CGRP assays

Our protocol was based on previous literature (Gavini et al., 2018). In particular, we chose to culture ganglia for 5–7 d to give the samples time to rebound from the injury caused by the acute dissection. Male mice (five to eight weeks) were deeply anesthetized with isoflurane before being decapitated and the nodose/jugular ganglia were quickly removed and stored in chilled HBSS (Invitrogen) on ice. The isolated ganglia were placed on a 30 mm, 0.4 μm pore size, hydrophilic Millicell culture insert (Millipore Sigma; catalog #PICM03050) and maintained on the insert in DMEM F-12 GlutaMax media (Invitrogen) supplemented with 20% heat-inactivated horse serum (Invitrogen, Life Technologies), and 1× penicillin streptomycin (Invitrogen). Cultures were maintained for 5–7 d with media changes every other day. After an overnight incubation in low serum (2.0%), cultures were stimulated with 500 ng/ml LPS or vehicle for 24 h before supernatant was collected. Supernatants were centrifuged to remove debris and loaded to a CGRP ELISA kit (Cayman Chemical; catalog #589001) and the protocol ran according to manufacturer’s instructions.

### Statistical analysis

All of the quantitative data are expressed as the mean value ± SEM. The numbers of mice per group are indicated in each figure. Statistical analysis was performed using GraphPad Prism software, version 6.07. As indicated in the legends, the data were analyzed using Student’s unpaired *t* test, one-way ANOVA or two-way ANOVA, followed by the *post hoc* test recommended by the software; *p* < 0.05 was considered statistically significant. Lower case letters were used to indicate groups found to be significantly different by the *post hoc* analysis. We also followed the journal’s recommendations in terms of data representations and estimation statistics (Ho et al., 2019). Estimation statistics are included in the legends of our main figures or Extended Data.

## Results

### Validation of a novel reactivable TLR4 mouse model

**Figure 1.**
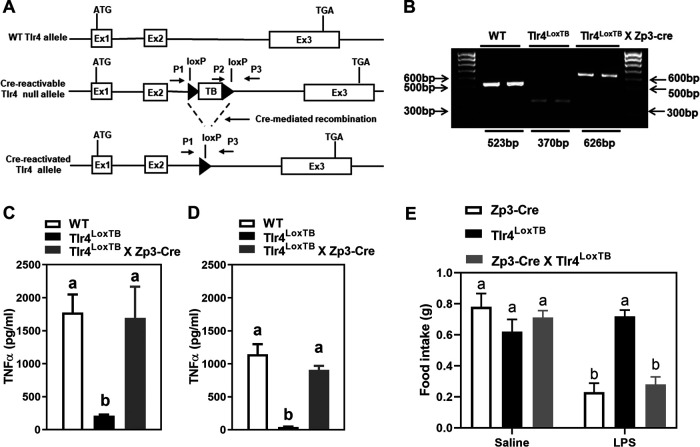
***A***, A loxP-flanked transcription-blocking cassette was inserted between exons 2 and 3 of the Tlr4 gene to generate mice lacking TLR4. ***B***, Genotyping analysis of tail genomic DNA from WT, Tlr4^LoxTB^, and Tlr4^LoxTB^ X Zp3-Cre mice. ***C***, ***D***, Plasma TNFα levels were measured in WT, Tlr4^LoxTB^ and Tlr4^LoxTB^ X Zp3-Cre mice (*n* = 3–5) 1.5 h after intraperitoneally injection of LPS with the concentration of either 1 mg/kg BW (***C***) or 0.5 mg/kg BW (***D***). In ***C***, data were analyzed using one-way ANOVA for genotype (*p* = 0.0013), followed by Dunnett’s test (WT vs Tlr4^LoxTB^ *p* = 0.0017). In ***D***, data were analyzed using one-way ANOVA for genotype (*p* = 0.0004), followed by Dunnett’s test (WT vs Tlr4^LoxTB^ *p* = 0.0003). For cytokines, letters denote significant differences between columns with *p* < 0.05, compared with corresponding Tlr4^LoxTB^ mice. ***E***, Graphs of 1-h cumulative food intake in response to saline or LPS (100 μg/kg, i.p.) in control ZP3Cre (white), Tlr4^LoxTB^ (black), and Zp3-Cre-reactivated mice (gray). Groups consisted of *n* = 5. Data are expressed as mean ± SEM and were analyzed using two-way ANOVA for genotype (*p* = 0.0116), treatment (*p* = 0.0002), and interaction (*p* = 0.001), followed by Sidak’s *post hoc* test. For each genotype, letters denote significant differences between columns with *p* < 0.05, compared with corresponding saline-treated mice. Estimation statistics was calculated in LPS-treated mice. Estimation statistics are included in Extended Data Figure 1-1.

We designed and generated a novel Tlr4 null (Tlr4^LoxTB^) mice whose Tlr4 expression is globally disrupted by a loxP-flanked TB cassette (LoxTB) that was inserted into the coding region of the Tlr4 gene (Fig. 1*A*). Crossing Tlr4^loxTB^ mice with Zp3-Cre mice produced mice in which expression of endogenous Tlr4 was globally reactivated in all cells (Fig. 1*A*,*B*). As a result, the LPS-induced inflammatory response in these mice was identical to WT mice (Fig. 1*C*,*D*). Specifically, the administration of LPS produced a not significantly different increase in circulating TNF-α in WT and reactivated animals. However, Tlr4^LoxTB^ mice (without Cre) behaved like global TLR4 knock-out mice and failed to respond to LPS (Fig. 1*C*,*D*). In a separate cohort, we tested the anorectic effects of a small dose of LPS (Fig. 1*E*). As anticipated, LPS suppressed food intake in Zp3-Cre mice, but not in Tlr4^LoxTB^ mice (without Cre). The anorectic effect of LPS (100 μg/kg, i.p.) was fully restored in mice with Tlr4 reactivated in all cells that normally express it (Fig. 1*E*). This is because the expression of Tlr4 remains driven by its endogenous promoter. These studies demonstrate the validity of our reactivable model.

10.1523/ENEURO.0254-20.2020.f1-1 Extended Data Figure 1-1 ***A***, ***B***, Estimation statistic corresponding to Figure 1*C*,*D*. Mean difference for two comparisons against the shared control WT are shown in the above Cumming estimation plot. The raw data are plotted on the upper axes. On the lower axes, mean differences are plotted as bootstrap sampling distributions. Each mean difference is depicted as a dot. Each 95% confidence interval is indicated by the ends of the vertical error bars. ***C***, Estimation statistic corresponding to Figure 1*E*. Mean difference for two comparisons against the shared control Zp3-Cre are shown in the above Cumming estimation plot. The raw data are plotted on the upper axes. On the lower axes, mean differences are plotted as bootstrap sampling distributions. Each mean difference is depicted as a dot. Each 95% confidence interval is indicated by the ends of the vertical error bars. Download Figure 1-1, TIF file.

### Tlr4-expressing vagal afferents do not initiate LPS-induced anorexia

**Figure 2.**
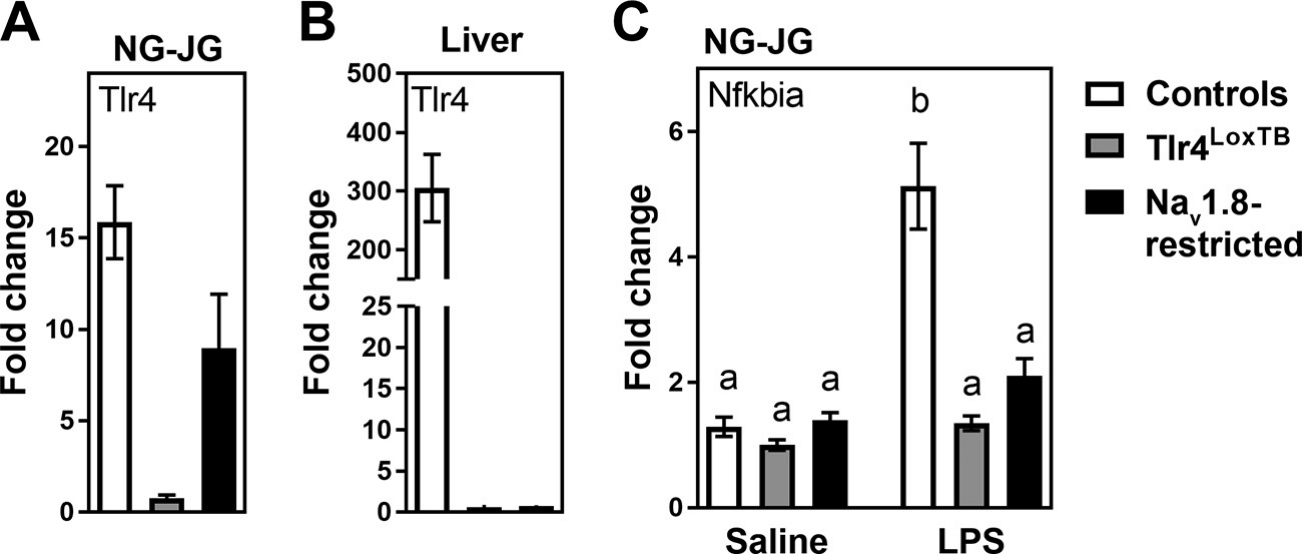
***A***, Tlr4 mRNA expression in the nodose-jugular ganglionic mass of control, Tlr4^LoxTB^, and Tlr4^LoxTB^ crossed with Na_v_1.8-Cre mice (Na_v_1.8-restricted TLR4). Our qPCR demonstrated the re-expression of Tlr4 in vagal afferents of reactivated animals. Groups consisted of *n* = 8–15 mice. ***B***, Restoration of Tlr4 expression was not observed in the liver of reactivated mice, demonstrating the specificity of the Cre-reactivated allele. Groups consisted of *n* = 3–7 mice. ***C***, Expression of Nfkbia mRNA in saline-treated and LPS-treated mice (2 mg/kg, i.p.) of each respective genotype. Groups consisted of *n* = 4–7 mice. Data were all obtained by qPCR, and 18S was used as a control gene. Data were analyzed using two-way ANOVA (treatment and genotype factors) followed by Sidak’s *post hoc* test. Letters denote significant differences between columns with *p* < 0.05. NG-JG: nodose-jugular ganglia; Nfkbia, NFκB inhibitor α. Estimation statistics are included in Extended Data Figure 2-1.

To test for functional TLR4 signaling in vagal afferents *in vivo*, we used the previously described Na_v_1.8-Cre line (Stirling et al., 2005) to generate cohorts of mice that endogenously express Tlr4 only in Na_v_1.8 neurons. By qPCR, we show that Tlr4 mRNA expression in the nodose-jugular ganglion was 15-fold higher in control mice than in Tlr4^LoxTB^ mice (without Cre; Fig. 2*A*). In Na_v_1.8-restricted Tlr4 animals, Tlr4 expression in the nodose-jugular ganglion was ∼8-fold higher than in Tlr4^LoxTB^ mice (Fig. 2*A*). Tlr4 was selectively re-expressed in Na_v_1.8 neurons, but not other Tlr4-expressing cell types. For example, Tlr4 was not reactivated in the liver, a tissue with high endogenous expression of Tlr4, but without Na_v_1.8-Cre cells (Fig. 2*B*). Lastly, the administration of LPS (2 mg/kg, i.p.) stimulated the expression of Nfkbia, a well-known TLR4 target gene (Quan et al., 1997), in the nodose-jugular ganglion of control animals (Fig. 2*C*). As anticipated, LPS did not elicit a transcriptional response in Tlr4^LoxTB^ mice (Fig. 2*C*). LPS also failed to significantly increase Nfkbia expression in the ganglia of Na_v_1.8-restricted Tlr4 mice (Fig. 2*C*). This result is consistent with the view that TLR ligands and cytokines are generally poor inducers of the NF-κB pathway in neurons compared with other cell types (Listwak et al., 2013). Overall, our data established the successful re-expression of Tlr4 in vagal afferents.

10.1523/ENEURO.0254-20.2020.f2-1 Extended Data Figure 2-1 ***A***, ***B***, Estimation statistic corresponding to Figure 2*A*,*B*. Mean difference for two comparisons against the shared controls are shown in the above Cumming estimation plot. The raw data are plotted on the upper axes. On the lower axes, mean differences are plotted as bootstrap sampling distributions. Each mean difference is depicted as a dot. Each 95% confidence interval is indicated by the ends of the vertical error bars. ***C***, Estimation statistic corresponding to Figure 2*C*. Estimation statistics was calculated in LPS-treated mice. The mean difference for two comparisons against the shared controls are shown in the above Cumming estimation plot. The raw data are plotted on the upper axes. On the lower axes, mean differences are plotted as bootstrap sampling distributions. Each mean difference is depicted as a dot. Each 95% confidence interval is indicated by the ends of the vertical error bars. Download Figure 2-1, TIF file.

**Figure 3.**
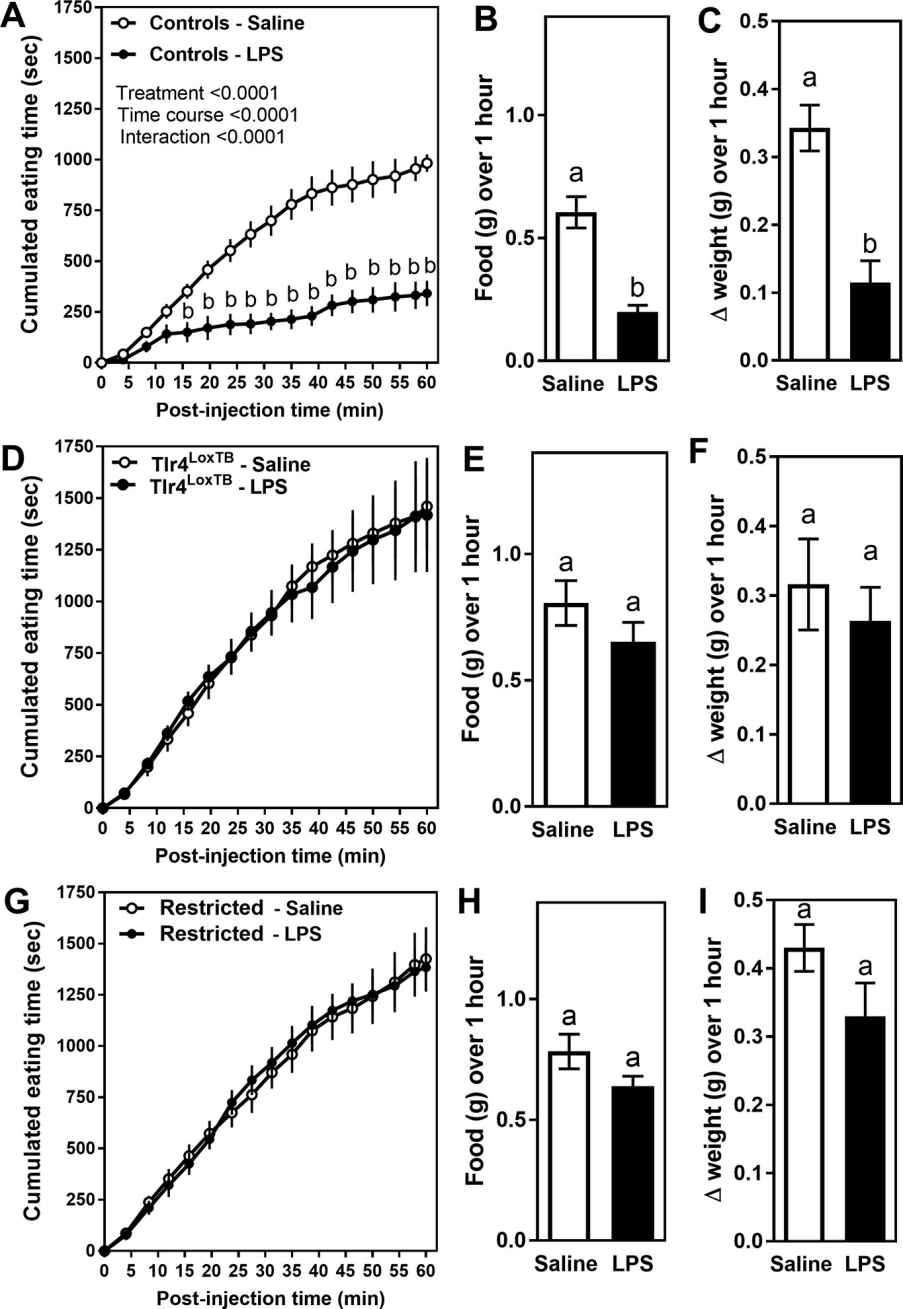
***A***, ***D***, ***G***, Graphs of the cumulative food intake in response to saline (white) or LPS (black; 100 μg/kg, i.p.) in control, Tlr4^LoxTB^, and Na_v_1.8-restricted TLR4 littermates. Groups consisted of *n* = 5–8 mice. Data were analyzed using two-way ANOVA (time and treatment) separately for each genotype, followed by Sidak’s *post hoc* test. The small letter b indicates points that are different from the saline-treated group. ***B***, ***E***, ***H***, Graph representing food intake or body weight change (***C***, ***F***, ***I***) over the hour following LPS or saline treatment. Data were analyzed using Student’s unpaired *t* test. Small letters indicate significant differences between columns. Data are all expressed as the mean values ± SEM over a period of 60 min. Estimation statistics are included in Extended Data Figure 3-1.

Early LPS-induced anorexia was investigated in control Na_v_1.8-Cre mice with intact Tlr4 expression. As anticipated, they showed rapid anorexia, starting ∼15 min following LPS injection (Fig. 3*A*). The total amount of food that LPS-treated mice ate over 1 h was also significantly reduced (Fig. 3*B*). Weight gain was significantly reduced in LPS-treated mice (Fig. 3*C*). In contrast, LPS did not elicit anorexia or weight loss in mice that completely lacked TLR4 expression (Fig. 3*D–F*). In mice expressing TLR4 only in Na_v_1.8 cells, LPS similarly failed to trigger anorexia or weight loss (Fig. 3*G*,*H*). We concluded that TLR4-bearing vagal Na_v_1.8 neurons do not contribute to the anorectic and cachectic actions of LPS at this particular dose and time point.

10.1523/ENEURO.0254-20.2020.f3-1 Extended Data Figure 3-1 ***A***, ***B***, Estimation statistic corresponding to Figure 3*B*,*C*. The mean difference between controls-saline and controls-LPS for food (***A***) and weight change (***B***) are shown in the Gardner–Altman estimation plot. Both groups are plotted on the left axes; the mean difference is plotted on a floating axis on the right as a bootstrap sampling distribution. The mean difference is depicted as a dot; the 95% confidence interval is indicated by the ends of the vertical error bar. ***C***, ***D***, Estimation statistic corresponding to Figure 3*E*,*F*. The mean difference between Tlr4LoxTB-Saline and Tlr4LoxTB-LPS for food (***A***) and weight change (***B***) are shown in the Gardner–Altman estimation plot. Both groups are plotted on the left axes; the mean difference is plotted on a floating axis on the right as a bootstrap sampling distribution. The mean difference is depicted as a dot; the 95% confidence interval is indicated by the ends of the vertical error bar. ***E***, ***F***, Estimation statistics corresponding to Figure 3*H*,*I*. The mean difference between restricted-saline and restricted-LPS for food (***E***) and weight change (***F***) are shown in the Gardner–Altman estimation plot. Both groups are plotted on the left axes; the mean difference is plotted on floating axes on the right as a bootstrap sampling distribution. The mean difference is depicted as a dot; the 95% confidence interval is indicated by the ends of the vertical error bar. Download Figure 3-1, TIF file.

### TLR4 is selectively enriched in CGRP-positive afferents

**Figure 4.**
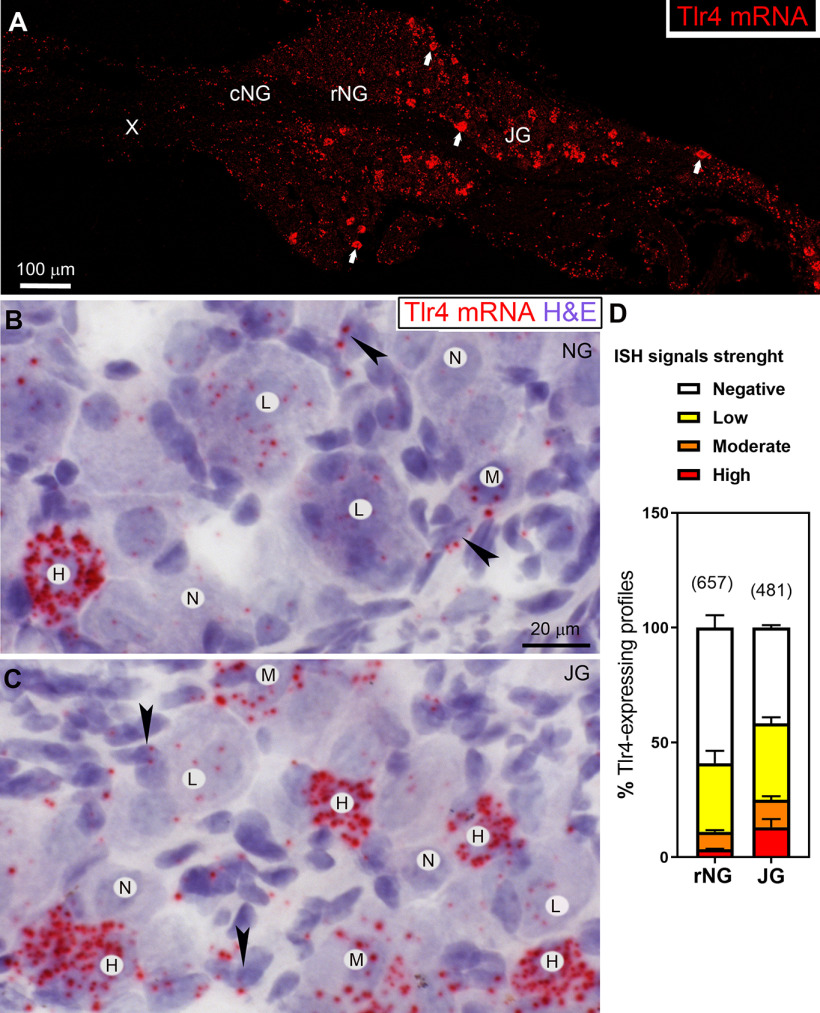
***A–D***, Chromogenic ISH for Tlr4 mRNA (red) in the nodose and jugular ganglia of C57Bl6/J mice. Using fluorescent illumination, scattered positive cells resembling neurons are observed mostly in the jugular portion of the ganglionic complex (white arrows). Under bright-field illumination, representative neuronal profiles were labeled as expressing Tlr4 signals at high (H), medium (M), or low levels (L). Cells with no signals were also apparent (N). Black arrows indicate putative non-neuronal cells surrounding neuronal profiles. Tissues were counterstained with hematoxylin (purple) to facilitate the identification of cellular profiles. The scale bars in ***B*** applies to ***C***. ***D***, Graph showing the percentage of neuronal profiles with different ISH signal strengths for Tlr4. Data are expressed as the mean percentage ± maximal values of Tlr4-expressing neurons in the nodose and jugular ganglia. The total number of counted profiles is indicated above each bar graph. The Extended Data Figure 4-1 includes a control of specificity for the Tlr4 probe. c, caudal; H, high intensity signal; HS, hematoxylin stain; M, medium intensity signal; N, no visible signal; cNG, caudal nodose ganglion; rNG, rostral nodose ganglion; JG, jugular ganglion; L, low intensity signal; r, rostral.

Although Tlr4 mRNA has previously been described in the nodose ganglion (Hosoi et al., 2005), little information is available regarding its exact cellular distribution. Chromogenic ISH allowed us to detect the signal for the Tlr4 transcript as a red precipitate (FastRed) visible under brightfield and fluorescent illuminations. The majority of the cellular profiles in the nodose and jugular ganglia was Tlr4-negative or contained very few Tlr4 signals (Fig. 4*A–D*). Diffuse and weak Tlr4 signals were seen in both neurons and non-neuronal cells in the nodose-jugular ganglia and the vagal trunk itself. However, a subset of cells resembling neurons contained moderate to high levels of Tlr4 signals (Fig. 4*A-D*). Notably, neurons with high expression levels were not uniformly distributed but were concentrated in the jugular ganglion and the rostral pole of the nodose ganglion (Fig. 4*A–D*). The caudal nodose ganglion itself, which contains a large population of vagal neurons including gastrointestinal afferents, was largely devoid of Tlr4-positive neurons. Based on our estimates, <12% of vagal afferents located in the rostral nodose ganglion proper contained moderate to high levels of Tlr4 signals (Fig. 4*D*). Considering that the boundary between the nodose and jugular-petrosal ganglia is ambiguous, many Tlr4-positive neurons observed in the nodose ganglion may have been part of the jugular ganglion. Indeed, over 50% (*n* = 3) of vagal afferents in the jugular ganglion were Tlr4-positive and close to 25% (*n* = 3) of them contained moderate to high levels of Tlr4.

10.1523/ENEURO.0254-20.2020.f4-1 Extended Data Figure 4-1 The specificity of the probe used to detect Tlr4 was validated by comparing by chromogenic ISH for Tlr4 mRNA (red) the ganglia obtained from mice with a globally reactivated allele Tlr4^LoxTB^ (***A***, ***B***) to that of Tlr4^LoxTB^ mice (***C***, ***D***). It is evident that robust red precipitates accumulated in Tlr4-positive neuronal profiles indicated by black arrows in reactivated mice. In contrast, the ganglion of Tlr4^LoxTB^ mice was almost entirely devoid of Tlr4 signals. Tissues were counterstained with hematoxylin (purple) and images were collected either under brightfield illumination. The scale bar in ***A*** applies to ***C***. JG, jugular ganglion. Download Figure 4-1, TIF file.

**Figure 5.**
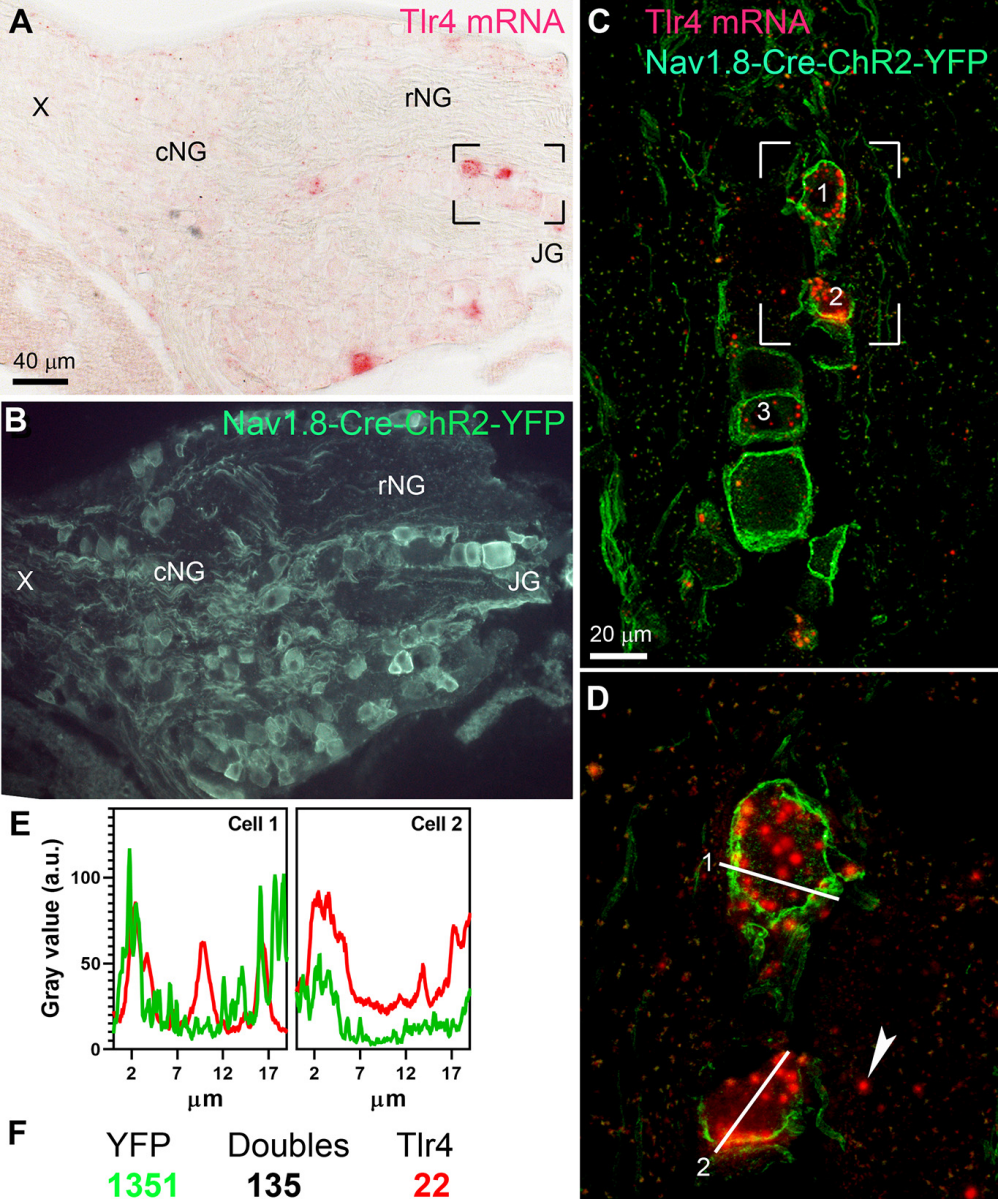
***A***, ***B***, Chromogenic ISH for Tlr4 mRNA (red) in the rostral nodose ganglion of the Na_v_1.8-Cre^ChR2-YFP^ mouse. Immunolabeling for GFP (green) was used to label the cell bodies of Na_v_1.8 neurons. ***C***, ***D***, At higher magnification (digital z-stack acquired by confocal microscopy), three large cells profiles with high intensity Tlr4 signals can be seen. The white arrowhead indicates scattered Tlr4 signal interpreted as a putative non-neuronal cell. ***E***, Plot profiles of the distribution across two cells represented in ***D***. The red and green lines correspond to the fluorescence intensity for Tlr4 and YFP, respectively. Notably, Tlr4 signals strongly accumulated within the boundary of ChR2-YFP-labeled neurons. ***F***, Absolute numbers of counted cell profiles. From these data, it was concluded that Tlr4 was primarily co-expressed by Na_v_1.8 neurons. cNG, caudal nodose ganglion; rNG, rostral nodose ganglion; JG, jugular ganglion; X, vagus nerve trunk.

**Figure 6.**
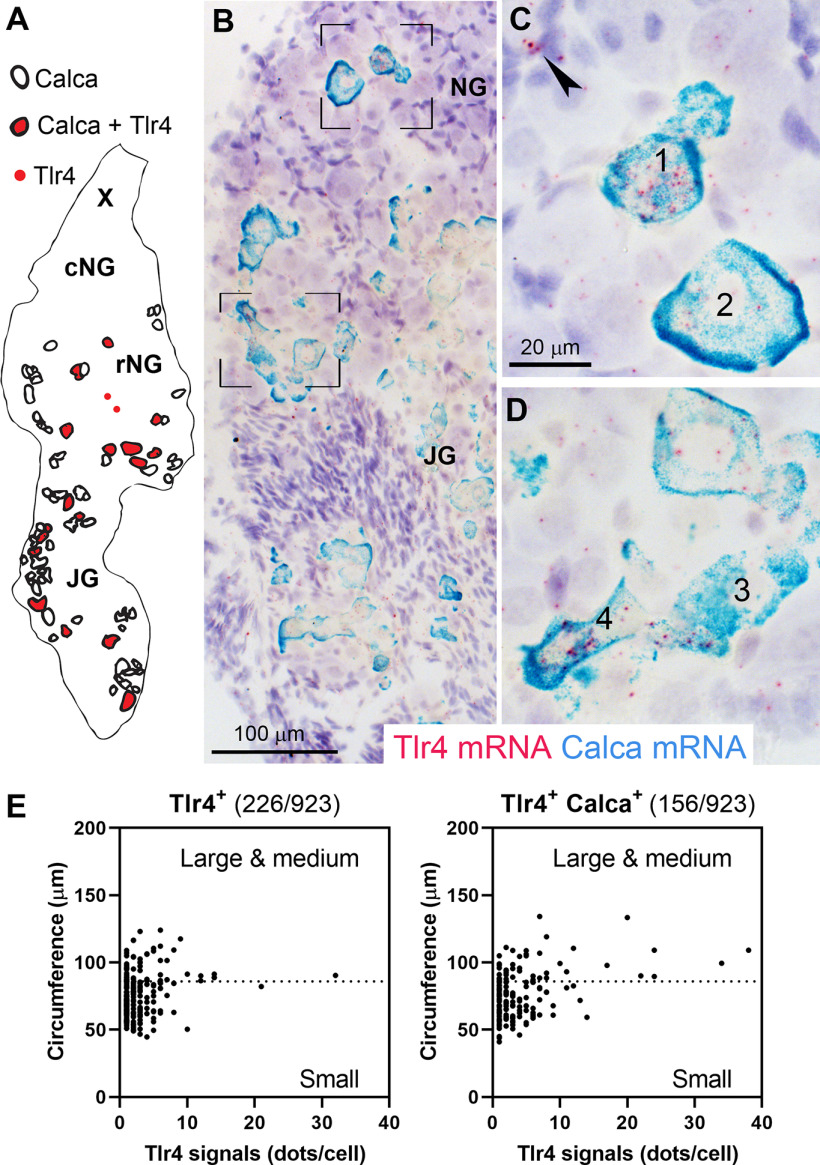
***A***, Camera lucida drawing of one vagal ganglionic complex processed for double chromogenic ISH for Tlr4 and Calca mRNAs. Each symbol represents one individual neuronal profile identified as positive for Tlr4 (red dots), Calca (open circles), or both signals (circles filled with red). Note that both Tlr4-positive and Calca-positive cells preferentially accumulated in the rostral pole of the ganglionic mass. ***B*–*D***, Representative bright-field images of doubly labeled neurons for Tlr4 (red) and Calca (blue) in the ganglionic mass of C57Bl6/J mice. Overall, these data indicate that Tlr4 and Calca were frequently co-expressed in the same neurons. The numbers are meant to indicate four examples of profiles co-expressing Calca and Tlr4 mRNAs. Ganglia were counterstained with hematoxylin. ***E***, Quantification of Tlr4 signals (dots) in cell profiles negative (left) or positive (right) for Calca. Individual cell profiles are displayed as black circles. A total of 923 cell counterstained profiles were counted, *n* = 4 mice. Using ImageJ, the circumference of each profile was determined and further used to categorize cell profiles into small or large/medium cells. Absolute numbers of tlr4-positive profiles is also indicated above each graph. Scale bar in ***C*** applies to ***D***. cNG, caudal nodose ganglion; rNG, rostral nodose ganglion; JG, jugular ganglion; X, vagus nerve trunk.

Our next studies sought to confirm the neuronal identity of Tlr4-expressing cells. Specifically, Na_v_1.8-Cre^ChR2-YFP^ was used to identify the cell bodies of vagal Na_v_1.8 neurons. Chromogenic ISH was combined with GFP immunohistochemistry to visualize neurons co-expressing Na_v_1.8 and Tlr4 (Fig. 5*A–F*). When combined with the detection of ChR2-labeled cells, it became evident that Tlr4 mRNA was expressed by Na_v_1.8 neurons (Fig. 5*D*,*E*). In the nodose-jugular ganglia, ∼84% (*n* = 4) of Tlr4-expressing cells were also Na_v_1.8-positive. Conversely, ∼9% (*n* = 4) of Na_v_1.8-positive neurons expressed Tlr4. The jugular ganglion and rostral nodose ganglion are known to contain vagal afferents producing CGRP (Helke and Niederer, 1990; Mazzone and Undem, 2016). Therefore, we next performed double chromogenic ISH to simultaneously detect Tlr4 and either Calca (CGRP precursor gene) or CGRP peptide. As anticipated, both Tlr4 and Calca-expressing neurons were the most abundant in the jugular ganglion and the most rostral portion of the nodose ganglion (Fig. 6*A–D*). Tlr4 mRNA signals often accumulated in Calca-expressing neurons (Fig. 6*B*,*C*), with an estimated 40% (*n* = 4) of Tlr4-expressing neurons also being Calca-positive (Fig. 6*E*). In summary, our anatomic data established that Tlr4 was predominantly, but not exclusively, expressed by a subset of vagal afferents producing Calca, which are known to be mainly airways and facial somatosensory and nociceptive afferents (Helke and Niederer, 1990; Mazzone and Undem, 2016).

**Figure 7.**
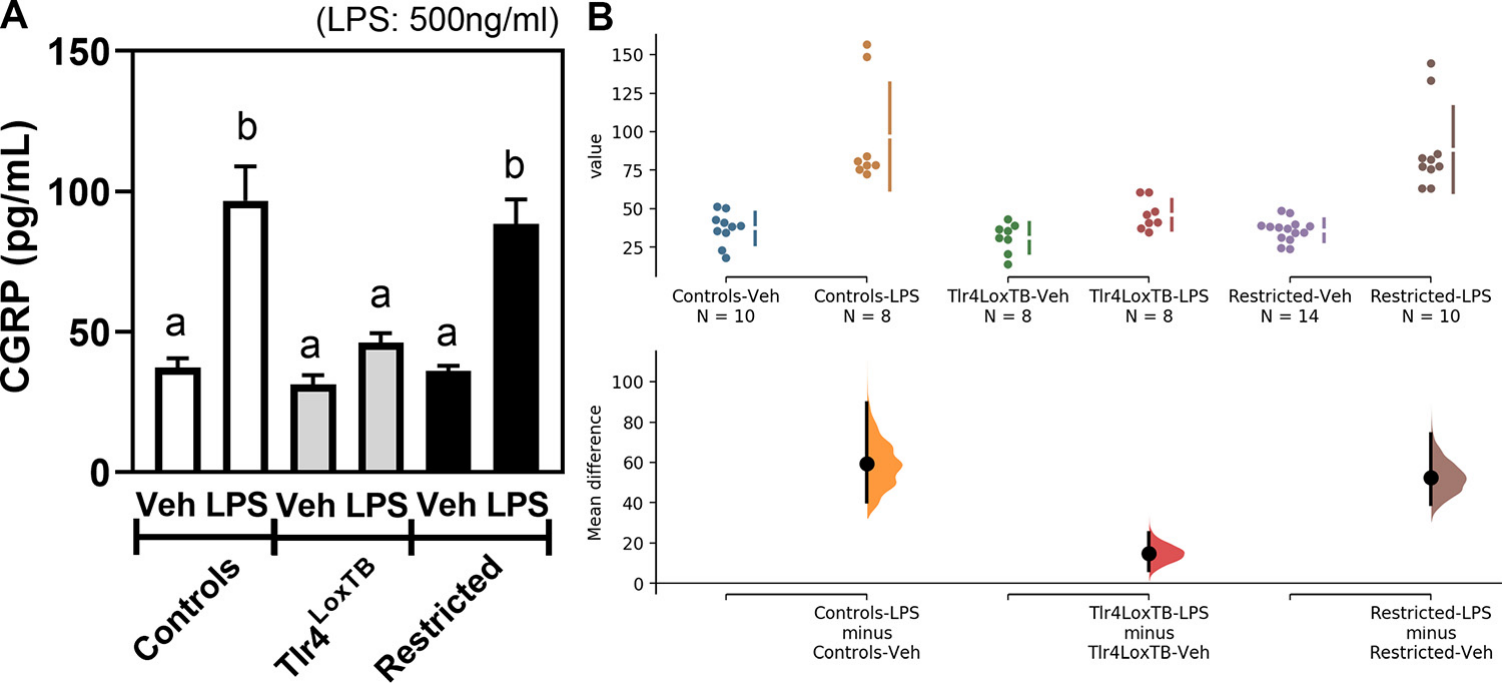
***A***, LPS-induced CGRP production from Tlr4-expressing vagal afferents. CGRP ELISA from nodose-jugular ganglia organotypic cultures isolated from WT (controls), Tlr4LoxTB (KO), and Tlr4^LoxTB^ X Na_v_1.8-Cre (restricted) animals and treated with LPS (500 ng/ml) or vehicle (sterile saline). Groups consists of ganglia from *n* = 5–7 animals. Data were analyzed using one-way ANOVA followed by Tukey’s *post hoc* test. Different letters indicate significant differences between columns. Data are all expressed as the mean values ± SEM. ***B***, Estimation statistics we included as the mean difference for three comparisons are shown in the Cumming estimation plot. The raw data are plotted on the upper axes; each mean difference is plotted on the lower axes as a bootstrap sampling distribution. Mean differences are depicted as dots; 95% confidence intervals are indicated by the ends of the vertical error bars. In Extended Data Figure 7-1, we further assessed the expression levels for Tlr4 and Calca in dorsal root ganglia cultured for 5 d.

CGRP is an immunomodulatory peptide released from peripheral afferents during bacterial infections (Lai et al., 2017). Thus, we tested whether TLR4 signaling is sufficient and/or required for the release of CGRP from cultured vagal afferents. As anticipated, the application of LPS robustly raised CGRP release (Fig. 7*A*,*B*). In Tlr4^LoxTB^ mice, no significant changes in CGRP release were noticed following LPS. In the ganglia of Na_v_1.8-restricted Tlr4 mice, the application of LPS raised CGRP release to the same degree as in the ganglia of control mice (Fig. 7*A*,*B*). These data demonstrated that TLR4 signaling in vagal afferents directly mediates CGRP release on LPS exposure. Of note, the application of LPS did not alter the transcription of Tlr4 and Calca in sensory ganglia cultured for 5 d (see Extended Data Figure 7-1).

10.1523/ENEURO.0254-20.2020.f7-1 Extended Data Figure 7-1 Q-PCR analysis using Taqman primers (see main text) of dorsal root ganglion (DRG) either freshly collected from WT mice or cultured for 5 d. In all samples, Tlr4 and Calca mRNAs were detected at moderate levels with averaged Ct values of 32. Notably, after 5 d in culture, levels of Tlr4 were robustly stimulated, but Calca remained unchanged. When LPS was applied as described in the main text, Tlr4 and Calca expression levels remained unchanged. Download Figure 7-1, TIF file.

## Discussion

### Novel tools for the study of TLR4

LPS is a potent inducer of proinflammatory molecules, a number of which share the same cellular targets and intracellular signaling pathways as TLR4 (Miyamoto and Verma, 1995; Muta et al., 2002; Skelly et al., 2013). Combined with the redundancy of cells expressing TLR4 throughout the body (Poltorak et al., 1998a; Lin et al., 2000; Laflamme and Rivest, 2001; Liu et al., 2002), this has made it particularly difficult to tease apart the cellular and molecular determinants of TLR4 actions. Here, we developed and validated a novel mouse model to manipulate TLR4 expression in a cell type-specific manner within the context of intact animals. In the current study, we used this model to investigate the role played by peripheral afferents in LPS-induced anorexia. In theory, this approach can be used to test TLR4 signaling in any genetically-targeted cells provided that a Cre line is available. It has also been difficult to study TLR4 actions because of the lack of validated anti-TLR4 antibodies. While radioisotopic ISH has been used to detect Tlr4-expressing cells in the CNS (Laflamme and Rivest, 2001; Chakravarty and Herkenham, 2005), the present study has refined these techniques to specifically analyze Tlr4 gene expression in vagal afferents. In particular, we used a highly sensitive ISH procedure called RNAscope (Advanced Cell Diagnostic; Wang et al., 2014; Grabinski et al., 2015). The latter technique allowed us to detect Tlr4 mRNA with virtually no background. It also allowed us to easily detect Tlr4 in combination with other transcripts or GFP.

### Role of vagal afferents during inflammatory anorexia

It is likely that peripheral afferents can rapidly convey information to the brain relevant to a potentially lethal threat, such as a Gram-negative bacterial infection. This phenomenon might possibly exist throughout the animal kingdom because sensory neurons in Aplysia sea slugs have been reported to be sensitive to bacterial LPS (Clatworthy and Grose, 1999). In rodents, numerous studies have suggested the ability of both vagal and spinal sensory neurons to respond to a broad range of inflammatory stimuli (Goehler et al., 1997; Ek et al., 1998; Ross et al., 2003; Eijkelkamp et al., 2010; La and Gebhart, 2011; Chiu et al., 2013). Prior studies have established that rodents with bilateral subdiaphragmatic surgical vagotomy showed diminished anorexia in response to LPS (Bret-Dibat et al., 1995; Sergeev and Akmaev, 2000). However, despite all of the evidence implicating peripheral afferents in sensing LPS, the requirement of vagal afferents in mediating LPS-induced anorexia has been difficult to demonstrate. For instance, other studies have reported identical degrees of LPS-induced anorexia in animals with capsaicin-mediated or surgery-mediated deafferentation (Schwartz et al., 1997; Porter et al., 1998b; Wieczorek et al., 2005). To our knowledge, prior *in vivo* studies have rarely examined LPS-induced anorexia before the 1-h postinjection time point (Kent et al., 1992; Liu et al., 2016). However, our data reinforced the view that anorexia is initiated rapidly after intraperitoneal LPS exposure. This observation led us to hypothesize that first, LPS may directly act on vagal afferents to suppress feeding; and second, that prior studies may have missed the early anorectic response to LPS. Contrary to expectation, our findings indicate that TLR4 signaling in peripheral afferents alone is not sufficient to initiate anorexia. As explained below, we believe these findings can be explained by the fact that TLR4 signaling occurs mostly in rostral nodose and jugular vagal afferents supplying the airways, rather than those involved in feeding. Instead, it is likely that LPS-induced anorexia is initiated by the *de novo* production of cytokines and prostaglandins within the brain (Yao et al., 1999; Layé et al., 2000; Sachot et al., 2004; Chakravarty and Herkenham, 2005; Ching et al., 2007; Elander et al., 2007; Rummel et al., 2008; Skelly et al., 2013). A recent single-cell sequencing study determined that the molecular make-up of jugular afferents is reminiscent of that of spinal nociceptors rather than vagal afferents of the nodose ganglion (Kupari et al., 2019). Thus, the difference of expression of Tlr4 mRNA in the nodose versus jugular ganglion is not completely surprising. Considering that the jugular and nodose ganglia are fused in rodents, one prior report of Tlr4 expression in the whole nodose ganglion may have been because of a jugular contamination (Hosoi et al., 2005).

### TLR4 signaling and vagal CGRP

In the CNS, TLR4 is enriched in non-neuronal cells, most notably including microglial cells (Lacroix et al., 1998; Laflamme and Rivest, 2001; Lehnardt et al., 2002; Olson and Miller, 2004; Chakravarty and Herkenham, 2005; Kigerl et al., 2007; Madore et al., 2013; Larochelle et al., 2015). Several laboratories also reported TLR4 expression in neurons (Rolls et al., 2007; Yoo et al., 2011; Leow-Dyke et al., 2012; Calvo-Rodríguez et al., 2017). However, the latter studies relied on *in vitro* data and/or unvalidated antibodies against TLR4. In contrast, high resolution ISH mapping studies have established that TLR4 expression was restricted to non-neuronal cells in normal, inflamed, and injured CNS (Laflamme and Rivest, 2001; Chakravarty and Herkenham, 2005; Kigerl et al., 2007). Hence, a general agreement is that CNS neurons do not express TLR4, or they do at very low levels. In contrast, TLR4 expression was found to be relatively high in a small subset of vagal afferents. Notably, we found that LPS did not significantly increase Nfκbia expression in Tlr4-expressing Na_v_1.8 neurons. This result is in agreement with the fact that LPS is a poor inducer of the NF-κB pathway in neurons compared with other cell types (Quan et al., 1997; Stern et al., 2000; Laflamme and Rivest, 2001; Chakravarty and Herkenham, 2005; Kigerl et al., 2007; Listwak et al., 2013). In addition, NFκB-independent signaling mechanisms may account for LPS actions on sensory neurons (Li et al., 2014; Meseguer et al., 2014; Allette et al., 2017; Boonen et al., 2018). This putative non-genomic effect of TLR4 on peripheral afferents differs from its canonical NFκB-dependent actions on immune, endocrine, and glial cells. Thus, additional studies are warranted in our Na_v_1.8-restricted TLR4 mice to elucidate LPS sensing mechanisms in vagal afferents. Considering the large amount of LPS that is constitutively contained within the gut lumen (Hersoug et al., 2016), we anticipated that TLR4 would be expressed by vagal afferents supplying the intestines. Based on our data, we cannot rule out entirely that certain vagal afferents innervating the gut expressed TLR4. In fact, a small subset of CGRP-positive vagal afferents was reported to supply the rodent stomach (Bai et al., 2019). Nonetheless, it must be stressed that Tlr4 mRNA was rarely seen in neurons of the caudal nodose ganglion, where reside vagal afferents innervating the gut. Thus, the common proposition that LPS signaling in vagal afferents sense gut microbial communities is not supported by our findings. Instead, we found abundant expression of TLR4 in CGRP-producing vagal afferents, a subgroup of vagal afferents supplying the upper airways, meninges, and facial skin (Mazzone and Undem, 2016). Moreover, our *in vitro* data indicate that TLR4 signaling is required and sufficient to mediate LPS-induced CGRP release. Interestingly, bacterial infections in the respiratory tract are common and can lead to sepsis more often than in any other primary sites of infection. CGRP is a well-known immuno-modulatory peptide released from vagal and spinal C-fiber endings (Myers et al., 1996; Abbadie et al., 2002; Abrahamsen et al., 2008; Chiu et al., 2013). Vagal afferents play a key role in airways defenses and coughing (Prescott et al., 2020; Ruhl et al., 2020). Therefore, it is tempting to speculate that the stimulation of TLR4 in vagal airway afferents plays an important immuno-modulatory role in the airways and lungs. For instance, a recent study demonstrated that the release of CGRP from respiratory vagal afferents is critical to bacterial clearance in lungs (Baral et al., 2018). Therefore, further studies in TLR4 reactivated mice are warranted to verify their susceptibility to respiratory infections. Our prediction is that the stimulation of TLR4-bearing vagal (and/or spinal) neurons supplying the airways during a Gram-negative bacterial infection would trigger an efferent response, probably involving the release of CGRP, aimed at containing inflammation and favoring bacterial clearance. It is without saying that molecules other than TLR4 may also be involved in mediating CGRP release during a bacterial infection, as suggested by several studies (Diogenes et al., 2011; Meseguer et al., 2014; Boonen et al., 2018). In particular, Meseguer and colleagues showed that the release of CGRP from isolated tracheas is a TRPA1-dependent phenomenon. However, based on data obtained with a Trpa1 global knock-out, it cannot be certain that neuronal TRPA1 was involved in the observed effects. Indeed, CGRP is also released from immune cells (Baliu-Piqué et al., 2014) and TRPA1 itself has been reported in immune and epithelial cells (Khalil et al., 2018). Moreover, the high dose of LPS used in this prior TRPA1-related study is believed to cause TLR4-independent effects because of LPS interacting with the membrane itself (Redeker and Briscoe, 2019; Yin et al., 2020). Using a lower dose of LPS, other authors have shown TRPA1 to be dispensable for LPS calcium imaging response in sensory neurons (Diogenes et al., 2011; Boonen et al., 2018). Once again, the advantage of our model is that we manipulated TLR4 only in Na_v_1.8 neurons of the nodose ganglion. In other words, it is likely that TLR4 and TRP channels are synergistically involved in the release of CGRP from neuronal endings, but TRP channels do not mediate this response alone. Lastly, it must be noted that the transcriptional profile of jugular vagal afferents resembles that of spinal and trigeminal nociceptors (Kupari et al., 2019). It is therefore likely that our findings may be extrapolated to other Tlr4-expressing peripheral nerves including spinal and trigeminal CGRP-positive afferents.

## Synthesis

Reviewing Editor: Quentin Pittman, University of Calgary Decisions are customarily a result of the Reviewing Editor and the peer reviewers coming together and discussing their recommendations until a consensus is reached. When revisions are invited, a fact-based synthesis statement explaining their decision and outlining what is needed to prepare a revision will be listed below. The following reviewer(s) agreed to reveal their identity: Karel Talavera, Cristian Acosta.

The view shared by both reviewers is that both the time course of TLR4 (particularly with regards to the actual levels of the receptor between the 2 systems) and a real quantitation using proper phenotypic markers for neuronal and non-neuronal cells are essential to lend credence to what you conclude. The antibody characterization can be saved if you can provide strong and convincing published data on its specificity. Please note also the comments with respect to the statistics as follows:

First, there is a complete lack of power calculations, so the size of the experimental groups seems a bit random (and it is reported vaguely, i.e. 3 to 7 mice, not indicating clearly how many exactly in each condition). Second, the data were not checked for normal distribution of the probability function (usind D’Agostino-Pearson, for example) and thus, we can not ascertain whether or not non-parametric tests would have been better suited to analyze the data. However, and given the small size of the groups, I would strongly expect that to be the case.

The actual reviews are appended below, each of which raises additional issues that must be addressed in the revision

Reviewer 1:

The main contribution of this study is the generation of mouse models that allow inquiring over the roles of TLR4 receptors in sensory neurons. It also sheds some light on the mechanisms underlying the effects of LPS, i.e., anorexia and CGRP release from sensory neurons.

1. Line 31, it is stated that “Toll-like receptor 4 (TLR4) is the receptor for lipopolysaccharides (LPS)”. This implies a picture that is too simplified, as actually MD-2 is the main LPS binding module of the TLR4-MD-2 receptor complex. In addition, as noted by the authors, several TRP channels are activated by LPS in a TLR4-independent manner (e.g., TRPA1, Meseguer et al. Nat Comm 2014 and TRPV4, Alpizar et al. Nat Comm 2017).

2. Lines 36-39, it is not clear why the fact that LPS triggers anorexic within minutes and that it does not cross the blood-brain barrier directly suggests that the implication of a peripheral vagal-mediated mechanism. The evidence for this comes in the next sentences.

3. Line 49, please avoid teleologic style, the preservation of a mechanism during evolution does not have any aim. See also line 348, it is not necessary to use a teleological point of view to describe biological phenomena.

4. Lines 56-58, none of the studies of Meseguer et al. and Boonen et al. contradict the functional expression of TLR4 in vagal afferents. These studies looked at intracellular Ca2+ in cultured DRG and nodose neurons and showed that some TRP channels can be activated by LPS, leading to acute pain and neurogenic inflammation in a TLR4-independent manner.

5. I would like to understand better this point, in lines 245 and 245 the authors state that “Crossing Tlr4loxTB mice with Zp3-Cre mice produced mice in which expression of endogenous Tlr4 was globally reactivated in all cells.” Is this in ALL cells of the body, even in those where no TLR4 is normally expressed?

6. In the same lines, please, specify how LPS was administered to induce the LPS-induced inflammatory response. Please, make sure that the details of LPS application are given for every experiment.

7. Lines 246 and 247, please change the use of “identical” to “not-significantly different”.

8. Line 250, specify how LPS and how much was administered.

9. Line 254, please write “To test for functional TLR4 signaling in vagal afferents in vivo”.

10. Line 257, delete “we showed that” or write it in present tense.

11. Lines 260-262, on what data do the authors support the statement “Higher Tlr4 levels in controls compared to Nav1.8-restricted Tlr4 mice were likely due to Tlr4 expression in non-neuronal cells.”

12. Line 262, write “show” instead of “showed", otherwise the reader may think that this was done in a previous study.

13. Lines 267-268, delete “More surprisingly,” and “However,” in line 269. A result is not surprising if the authors can already advance an explanation from previous studies.

14. Lines 271-272, it is not clear what data confirms the neuronal identity of Tlr4-expressing cells in the nodose ganglion. In fact, the sentence in lines 260-262 “Higher Tlr4 levels in controls compared to Nav1.8-restricted Tlr4 mice were likely due to Tlr4 expression in non-neuronal cells.” seems to contradict this statement.

15. Lines 272-273, it is claimed that the authors “established the successful re-expression of Tlr4 in vagal afferents” but only ganglia were tested for expressing, not nerve fibers.

16. Line 308, it is stated that “approximately 84% (n=4) of Tlr4-expressing cells were also Nav1.8-positive”. Isn’t the other 16% negative for Nav1.8, contradicting another statement given below “we never observed Tlr4-expressing neurons that were Nav1.8-negative”?

17. This co-expression descriptions should be backed-up with actual numbers of neurons expressing the different markers.

18. Lines 315-317, the finding that “an estimated 38% (n=3) of Calca-expressing neurons also being Tlr4-positive” does not tell about how much Tlr4 mRNA signals preferentially accumulate in Calca-expressing neurons, but vice versa.

19. Lines 319-320, the data provided does not show that as claimed “Tlr4 was mainly expressed by a small subset of vagal afferent C-fibers producing CGRP”. Please, note also that the co-expression study was performed at the level of the ganglia, not at the level of afferent C-fibers.

20. It is recommended that Venn diagrams are used for better representation of all co-expression data.

21. Line 322, delete “know”.

22. Line 324, please note that the CGRP release determinations were not performed in “cultured vagal afferents” but in cultured ganglia. For the same reason the conclusion given in lines 328-329 about vagal afferents is not supported.

23. It is unclear why the no reference is made to the results of Meseguer et al. (Nat Comm., 2014) showing that the CGRP release triggered by LPS from isolated mouse trachea was entirely dependent on TRPA1.

Reviewer 2:

The main advance is the generation and validation of a new transgenic mouse model that could be helpful to study the role of TLR4 in a subpopulation of neurons expressing Nav1.8 (albeit the authors did not do a characterization of the expression of the receptor in either DRG or trigeminal neurons, the other sites of high Nav1.8 expression). In terms of the role of TLR4 as mediator in the release of CGRP, the results are not that novel (others have shown that LPS acting via TLR4/MD2 can trigger the release of neuropeptides from DRG, nnodose and trigeminal neurons). As the authors postulates a role for this loop of LPS-TLR4-CGRP in nodose and jugular neurons in fighting airway infections but provide no proof of that, the advance to the field is present but it is minimal (barely incremental).

The starting point of the study was to examine the involvement of TLR4 expressed by Nav1.8+ neurons in LPS stimulated rapid-onset anorexia. This rendered a surprisingly negative result. The authors decided to study the expression pattern of TLR4 in vagal afferents other than those involved in innervating the gut and ended up discovering a subtype of TLR4+ Nav1.8+ CGRP+ neurons that release CGRP when exposed to LPS.

They also present data that validate a new transgenic mouse model of reactivable TLR4, that can make a valuable contribution to further studies that focus on Nav1.8-expressing neurons and the role that TLR4 may have on them.

This is a simple, straight forward study. My concerns follows:

1. As a general comment, please refrain from stating that all Nav1.8-expressing neurons are unmyelinated as this is clearly not the case (Djouhri et al., 2003). In fact, Nav1.8 is expressed significantly in nociceptive myelinated A-fibers.

2. In the Significance Statement the authors state that their findings could explain “How nerves modulate bacterial infections”. But I wonder, is it not the other way around? The bacterial infection presumably triggers the release of CGRP via activation of TLR4, thus in any event it is a bidirectional phenomenon.

3. A diagram explaining the experimental design would aid the presentation of the different experimental procedures carried out in this study.

4. qPCR must be reported in full - and the authors must abide by the standards of publication using this technique, particularly as they used the ΔΔ2CT method (Bustin et al., 2010). This means providing data on efficiency curves, melting curves and the sequences for all primers used, plus how quality of the mRNA was determined, how much of total RNA was used, etc.

5. Positive and negative controls for the immunostaining are missing and must be included to appreciate the real magnitude of the background staining. Full characterization of the anti-CGRP antibody must also be included.

6. What intensity was considered to label a cell as “positively stained”? How was that intensity determined? How many sections were analyzed and from how many animals? How many images per ganglia? This must be disclosed in full.

7. Why were ganglia cultured for 5 to 7 days? The authors must provide a convincing argument to do this as time in culture profoundly alters the phenotype of the neurons, among other parameters. Proof that TLR4 is not down regulated or upregulated by the culture conditions must be provided. Same goes for CGRP levels. What about measuring the ability of LPS to trigger the release of CGRP in acutely dissociated neurons? Or in their same organotipic preparation but at shorter times?

8. The study lacks power calculations.Also, normal distribution of probability for each data set should have been performed. Non-parametric tests are often better suited to compare data with non-normal distributions.

9. Markers of neurons and non-neuronal cells would have been far more convincing. Also some other positive tissue with high levels of TLR4 should have been used for comparison. In 5C there are some accumulations of signal suggesting at least some mRNA for TLR4 is still present despite the genetic suppression. Is it in non-neuronal cells? This should be demonstrated.

10. The quantitative approach of the authors to report the proportions and numbers of neurons or cells expressing TLR4 (or calca) mRNA is not convincing enough - albeit the authors state that they are reporting only estimations. However, I believe they can do much better and do a proper measurement, with objective rather than subjective measurements.It would be interesting to see lower magnification images of the contents shown in Figure 6.

Minor comments

Lines 307 to 310: The authors state that 84% of TLR4-expressing neurons were Nav1.8+. Therefore there were some TLR4-expressing neurons that were negative for Nav1.8. This needs to be corrected.

Please add the dose of LPS in plots 1C and 1D. Sample sizes are variable in 1C with some of the columns representing data from just 3 mice. Non parametric statistics should have been used in that case. I would also prefer to see scatter plots to have an idea of how homogeneous the levels of TNF-a are.

Correct the statement in line 322.

## References

[B1] Abbadie C, Lombard MC, Besson JM, Trafton JA, Basbaum AI (2002) Mu and delta opioid receptor-like immunoreactivity in the cervical spinal cord of the rat after dorsal rhizotomy or neonatal capsaicin: an analysis of pre- and postsynaptic receptor distributions. Brain Res 930:150–162. 10.1016/s0006-8993(02)02242-4 11879805

[B2] Abrahamsen B, Zhao J, Asante CO, Cendan CM, Marsh S, Martinez-Barbera JP, Nassar MA, Dickenson AH, Wood JN (2008) The cell and molecular basis of mechanical, cold, and inflammatory pain. Science 321:702–705. 10.1126/science.1156916 18669863

[B3] Allette YM, Kim Y, Randolph AL, Smith JA, Ripsch MS, White FA (2017) Decoy peptide targeted to Toll-IL-1R domain inhibits LPS and TLR4-active metabolite morphine-3 glucuronide sensitization of sensory neurons. Sci Rep 7:3741. 10.1038/s41598-017-03447-9 28623271 PMC5473853

[B4] Alpizar YA, Boonen B, Sanchez A, Jung C, López-Requena A, Naert R, Steelant B, Luyts K, Plata C, De Vooght V, Vanoirbeek JAJ, Meseguer VM, Voets T, Alvarez JL, Hellings PW, Hoet PHM, Nemery B, Valverde MA, Talavera K (2017) TRPV4 activation triggers protective responses to bacterial lipopolysaccharides in airway epithelial cells. Nat Commun 8:1059. 10.1038/s41467-017-01201-3 29057902 PMC5651912

[B5] Bai L, Mesgarzadeh S, Ramesh KS, Huey EL, Liu Y, Gray LA, Aitken TJ, Chen Y, Beutler LR, Ahn JS, Madisen L, Zeng H, Krasnow MA, Knight ZA (2019) Genetic identification of vagal sensory neurons that control feeding. Cell 179:1129–1143.e3. 10.1016/j.cell.2019.10.031 31730854 PMC6916730

[B6] Baliu-Piqué M, Jusek G, Holzmann B (2014) Neuroimmunological communication via CGRP promotes the development of a regulatory phenotype in TLR4-stimulated macrophages. Eur J Immunol 44:3708–3716. 10.1002/eji.201444553 25316186

[B7] Banks WA, Robinson SM (2010) Minimal penetration of lipopolysaccharide across the murine blood-brain barrier. Brain Behav Immun 24:102–109. 10.1016/j.bbi.2009.09.001 19735725 PMC2789209

[B8] Baral P, Umans BD, Li L, Wallrapp A, Bist M, Kirschbaum T, Wei Y, Zhou Y, Kuchroo VK, Burkett PR, Yipp BG, Liberles SD, Chiu IM (2018) Author correction: nociceptor sensory neurons suppress neutrophil and gammadelta T cell responses in bacterial lung infections and lethal pneumonia. Nat Med 24:417–426. 10.1038/nm.4501 29505031 PMC6263165

[B9] Berglund ED, Vianna CR, Donato J Jr, Kim MH, Chuang JC, Lee CE, Lauzon DA, Lin P, Brule LJ, Scott MM, Coppari R, Elmquist JK (2012) Direct leptin action on POMC neurons regulates glucose homeostasis and hepatic insulin sensitivity in mice. J Clin Invest 122:1000–1009. 10.1172/JCI59816 22326958 PMC3287225

[B10] Bluthé RM, Walter V, Parnet P, Layé S, Lestage J, Verrier D, Poole S, Stenning BE, Kelley KW, Dantzer R (1994) Lipopolysaccharide induces sickness behaviour in rats by a vagal mediated mechanism. C R Acad Sci III 317:499–503. 7987701

[B11] Boonen B, Alpizar YA, Sanchez A, López-Requena A, Voets T, Talavera K (2018) Differential effects of lipopolysaccharide on mouse sensory TRP channels. Cell calcium 73:72–81. 10.1016/j.ceca.2018.04.004 29689522

[B12] Bret-Dibat JL, Dantzer R (2000) Cholecystokinin receptors do not mediate the suppression of food-motivated behavior by lipopolysaccharide and interleukin-1 beta in mice. Physiol Behav 69:325–331. 10.1016/S0031-9384(00)00212-2 10869599

[B13] Bret-Dibat JL, Bluthé RM, Kent S, Kelley KW, Dantzer R (1995) Lipopolysaccharide and interleukin-1 depress food-motivated behavior in mice by a vagal-mediated mechanism. Brain Behav Immun 9:242–246. 10.1006/brbi.1995.1023 8590821

[B14] Bret-Dibat JL, Creminon C, Couraud JY, Kelley KW, Dantzer R, Kent S (1997) Systemic capsaicin pretreatment fails to block the decrease in food-motivated behavior induced by lipopolysaccharide and interleukin-1beta. Brain Res Bull 42:443–449. 10.1016/s0361-9230(96)00370-x 9128919

[B15] Calvo-Rodríguez M, de la Fuente C, García-Durillo M, García-Rodríguez C, Villalobos C, Núñez L (2017) Aging and amyloid beta oligomers enhance TLR4 expression, LPS-induced Ca(2+) responses, and neuron cell death in cultured rat hippocampal neurons. J Neuroinflammation 14:24. 10.1186/s12974-017-0802-0 28143556 PMC5282876

[B16] Chakravarty S, Herkenham M (2005) Toll-like receptor 4 on nonhematopoietic cells sustains CNS inflammation during endotoxemia, independent of systemic cytokines. J Neurosci 25:1788–1796. 10.1523/JNEUROSCI.4268-04.2005 15716415 PMC6725921

[B17] Ching S, Zhang H, Belevych N, He L, Lai W, Pu XA, Jaeger LB, Chen Q, Quan N (2007) Endothelial-specific knockdown of interleukin-1 (IL-1) type 1 receptor differentially alters CNS responses to IL-1 depending on its route of administration. J Neurosci 27:10476–10486. 10.1523/JNEUROSCI.3357-07.2007 17898219 PMC6673171

[B18] Chiu IM, Heesters BA, Ghasemlou N, Von Hehn CA, Zhao F, Tran J, Wainger B, Strominger A, Muralidharan S, Horswill AR, Bubeck Wardenburg J, Hwang SW, Carroll MC, Woolf CJ (2013) Bacteria activate sensory neurons that modulate pain and inflammation. Nature 501:52–57. 10.1038/nature12479 23965627 PMC3773968

[B19] Chow JC, Young DW, Golenbock DT, Christ WJ, Gusovsky F (1999) Toll-like receptor-4 mediates lipopolysaccharide-induced signal transduction. J Biol Chem 274:10689–10692. 10.1074/jbc.274.16.10689 10196138

[B20] Clatworthy AL, Grose E (1999) Immune-mediated alterations in nociceptive sensory function in *Aplysia californica*. J Exp Biol 202:623–630. 9929463 10.1242/jeb.202.5.623

[B21] Daniel JA, Whitlock BK, Marks DL, Gard JA, Sartin JL (2016) Leukemia inhibitory factor as a mediator of lipopolysaccharide effects on appetite and selected hormones and metabolites. J Anim Sci 94:2789–2797. 10.2527/jas.2016-0396 27482666

[B22] de Lartigue G, Barbier de la Serre C, Espero E, Lee J, Raybould HE (2011) Diet-induced obesity leads to the development of leptin resistance in vagal afferent neurons. Am J Physiol Endocrinol Metab 301:E187–E195. 10.1152/ajpendo.00056.2011 21521717 PMC3129833

[B23] Diogenes A, Ferraz CC, Akopian AN, Henry MA, Hargreaves KM (2011) LPS sensitizes TRPV1 via activation of TLR4 in trigeminal sensory neurons. J Dent Res 90:759–764. 10.1177/0022034511400225 21393555

[B24] Due MR, Piekarz AD, Wilson N, Feldman P, Ripsch MS, Chavez S, Yin H, Khanna R, White FA (2012) Neuroexcitatory effects of morphine-3-glucuronide are dependent on Toll-like receptor 4 signaling. J Neuroinflammation 9:200. 10.1186/1742-2094-9-200 22898544 PMC3519737

[B25] Eijkelkamp N, Heijnen CJ, Willemen HL, Deumens R, Joosten EA, Kleibeuker W, den Hartog IJ, van Velthoven CT, Nijboer C, Nassar MA, Dorn GW 2nd, Wood JN, Kavelaars A (2010) GRK2: a novel cell-specific regulator of severity and duration of inflammatory pain. J Neurosci 30:2138–2149. 10.1523/JNEUROSCI.5752-09.2010 20147541 PMC3129713

[B26] Ek M, Kurosawa M, Lundeberg T, Ericsson A (1998) Activation of vagal afferents after intravenous injection of interleukin-1beta: role of endogenous prostaglandins. J Neurosci 18:9471–9479. 9801384 10.1523/JNEUROSCI.18-22-09471.1998 PMC6792875

[B27] Elander L, Engström L, Hallbeck M, Blomqvist A (2007) IL-1beta and LPS induce anorexia by distinct mechanisms differentially dependent on microsomal prostaglandin E synthase-1. Am J Physiol Regul Integr Comp Physiol 292:R258–267. 10.1152/ajpregu.00511.2006 16946079

[B28] Essner RA, Smith AG, Jamnik AA, Ryba AR, Trutner ZD, Carter ME (2017) AgRP neurons can increase food intake during conditions of appetite suppression and inhibit anorexigenic parabrachial neurons. J Neurosci 37:8678–8687. 10.1523/JNEUROSCI.0798-17.2017 28821663 PMC5588461

[B29] Feldman-Goriachnik R, Belzer V, Hanani M (2015) Systemic inflammation activates satellite glial cells in the mouse nodose ganglion and alters their functions. Glia 63:2121–2132. 10.1002/glia.22881 26109245

[B30] Gavini CK, Bookout AL, Bonomo R, Gautron L, Lee S, Mansuy-Aubert V (2018) Liver X receptors protect dorsal root ganglia from obesity-induced endoplasmic reticulum stress and mechanical allodynia. Cell Rep 25:271–277.e4. 10.1016/j.celrep.2018.09.046 30304667 PMC7732131

[B31] Goehler LE, Relton JK, Dripps D, Kiechle R, Tartaglia N, Maier SF, Watkins LR (1997) Vagal paraganglia bind biotinylated interleukin-1 receptor antagonist: a possible mechanism for immune-to-brain communication. Brain Res Bull 43:357–364. 10.1016/s0361-9230(97)00020-8 9227848

[B32] Grabinski TM, Kneynsberg A, Manfredsson FP, Kanaan NM (2015) A method for combining RNAscope in situ hybridization with immunohistochemistry in thick free-floating brain sections and primary neuronal cultures. PLoS One 10:e0120120. 10.1371/journal.pone.0120120 25794171 PMC4368734

[B33] Grossberg AJ, Scarlett JM, Zhu X, Bowe DD, Batra AK, Braun TP, Marks DL (2010) Arcuate nucleus proopiomelanocortin neurons mediate the acute anorectic actions of leukemia inhibitory factor via gp130. Endocrinology 151:606–616. 10.1210/en.2009-1135 20016025 PMC2817620

[B34] Hansen MK, Daniels S, Goehler LE, Gaykema RP, Maier SF, Watkins LR (2000) Subdiaphragmatic vagotomy does not block intraperitoneal lipopolysaccharide-induced fever. Auton Neurosci 85:83–87. 10.1016/S1566-0702(00)00224-1 11189031

[B35] Helke CJ, Niederer AJ (1990) Studies on the coexistence of substance P with other putative transmitters in the nodose and petrosal ganglia. Synapse 5:144–151. 10.1002/syn.890050209 1689873

[B36] Helley MP, Abate W, Jackson SK, Bennett JH, Thompson SW (2015) The expression of Toll-like receptor 4, 7 and co-receptors in neurochemical sub-populations of rat trigeminal ganglion sensory neurons. Neuroscience 310:686–698. 10.1016/j.neuroscience.2015.09.069 26434622

[B37] Hersoug LG, Møller P, Loft S (2016) Gut microbiota-derived lipopolysaccharide uptake and trafficking to adipose tissue: implications for inflammation and obesity. Obes Rev 17:297–312. 10.1111/obr.12370 26712364

[B38] Ho J, Tumkaya T, Aryal S, Choi H, Claridge-Chang A (2019) Moving beyond P values: data analysis with estimation graphics. Nat Methods 16:565–566. 10.1038/s41592-019-0470-3 31217592

[B39] Hoshino K, Takeuchi O, Kawai T, Sanjo H, Ogawa T, Takeda Y, Takeda K, Akira S (1999) Cutting edge: toll-like receptor 4 (TLR4)-deficient mice are hyporesponsive to lipopolysaccharide: evidence for TLR4 as the Lps gene product. J Immunol 162:3749–3752. 10201887

[B40] Hosoi T, Okuma Y, Matsuda T, Nomura Y (2005) Novel pathway for LPS-induced afferent vagus nerve activation: possible role of nodose ganglion. Auton Neurosci 120:104–107. 10.1016/j.autneu.2004.11.012 15919243

[B41] Hua XY, Chen P, Fox A, Myers RR (1996) Involvement of cytokines in lipopolysaccharide-induced facilitation of CGRP release from capsaicin-sensitive nerves in the trachea: studies with interleukin-1beta and tumor necrosis factor-alpha. J Neurosci 16:4742–4748. 8764661 10.1523/JNEUROSCI.16-15-04742.1996 PMC6579029

[B42] Huang HY, Lai YL (2003) Lipopolysaccharide induces preprotachykinin gene expression. Am J Respir Cell Mol Biol 29:606–612. 10.1165/rcmb.2002-0107OC 12738685

[B43] Kent S, Kelley KW, Dantzer R (1992) Effects of lipopolysaccharide on food-motivated behavior in the rat are not blocked by an interleukin-1 receptor antagonist. Neurosci Lett 145:83–86. 10.1016/0304-3940(92)90209-P 1461574

[B44] Khalil M, Alliger K, Weidinger C, Yerinde C, Wirtz S, Becker C, Engel MA (2018) Functional Role of Transient Receptor Potential Channels in Immune Cells and Epithelia. Front Immunol 9:174. 10.3389/fimmu.2018.00174 29467763 PMC5808302

[B45] Kigerl KA, Lai W, Rivest S, Hart RP, Satoskar AR, Popovich PG (2007) Toll-like receptor (TLR)-2 and TLR-4 regulate inflammation, gliosis, and myelin sparing after spinal cord injury. J Neurochem 102:37–50. 10.1111/j.1471-4159.2007.04524.x 17403033

[B46] Konsman JP, Luheshi GN, Bluthé RM, Dantzer R (2000) The vagus nerve mediates behavioural depression, but not fever, in response to peripheral immune signals; a functional anatomical analysis. Eur J Neurosci 12:4434–4446. 10.1046/j.0953-816x.2000.01319.x 11122354

[B47] Kunda PE, Cavicchia JC, Acosta CG (2014) Lipopolysaccharides and trophic factors regulate the LPS receptor complex in nodose and trigeminal neurons. Neuroscience 280:60–72. 10.1016/j.neuroscience.2014.08.053 25218806

[B48] Kupari J, Häring M, Agirre E, Castelo-Branco G, Ernfors P (2019) An atlas of vagal sensory neurons and their molecular specialization. Cell Rep 27:2508–2523.e4. 10.1016/j.celrep.2019.04.096 31116992 PMC6533201

[B49] La JH, Gebhart GF (2011) Colitis decreases mechanosensitive K2P channel expression and function in mouse colon sensory neurons. Am J Physiol Gastrointest Liver Physiol 301:G165–G174. 10.1152/ajpgi.00417.2010 21512155 PMC3129930

[B50] Lacroix S, Feinstein D, Rivest S (1998) The bacterial endotoxin lipopolysaccharide has the ability to target the brain in upregulating its membrane CD14 receptor within specific cellular populations. Brain Pathol 8:625–640. 10.1111/j.1750-3639.1998.tb00189.x 9804372 PMC8098216

[B51] Laflamme N, Rivest S (2001) Toll-like receptor 4: the missing link of the cerebral innate immune response triggered by circulating gram-negative bacterial cell wall components. FASEB J 15:155–163. 10.1096/fj.00-0339com 11149903

[B52] Lai NY, Mills K, Chiu IM (2017) Sensory neuron regulation of gastrointestinal inflammation and bacterial host defense. J Intern Med 282:5–23. 10.1111/joim.12591 28155242 PMC5474171

[B53] Larochelle A, Bellavance MA, Rivest S (2015) Role of adaptor protein MyD88 in TLR-mediated preconditioning and neuroprotection after acute excitotoxicity. Brain Behav Immun 46:221–231. 10.1016/j.bbi.2015.02.019 25733102

[B54] Lawrence CB, Brough D, Knight EM (2012) Obese mice exhibit an altered behavioural and inflammatory response to lipopolysaccharide. Dis Model Mech 5:649–659. 10.1242/dmm.009068 22328591 PMC3424462

[B55] Layé S, Parnet P, Goujon E, Dantzer R (1994) Peripheral administration of lipopolysaccharide induces the expression of cytokine transcripts in the brain and pituitary of mice. Brain Res Mol Brain Res 27:157–162. 10.1016/0169-328x(94)90197-x 7877446

[B56] Layé S, Bluthé RM, Kent S, Combe C, Médina C, Parnet P, Kelley K, Dantzer R (1995) Subdiaphragmatic vagotomy blocks induction of IL-1 beta mRNA in mice brain in response to peripheral LPS. Am J Physiol 268:R1327–R1331. 10.1152/ajpregu.1995.268.5.R1327 7771597

[B57] Layé S, Gheusi G, Cremona S, Combe C, Kelley K, Dantzer R, Parnet P (2000) Endogenous brain IL-1 mediates LPS-induced anorexia and hypothalamic cytokine expression. Am J Physiol Regul Integr Comp Physiol 279:R93–98. 10.1152/ajpregu.2000.279.1.R93 10896869

[B58] Lee EC, Yu D, Martinez de Velasco J, Tessarollo L, Swing DA, Court DL, Jenkins NA, Copeland NG (2001) A highly efficient *Escherichia coli*-based chromosome engineering system adapted for recombinogenic targeting and subcloning of BAC DNA. Genomics 73:56–65. 10.1006/geno.2000.6451 11352566

[B59] Lehnardt S, Lachance C, Patrizi S, Lefebvre S, Follett PL, Jensen FE, Rosenberg PA, Volpe JJ, Vartanian T (2002) The toll-like receptor TLR4 is necessary for lipopolysaccharide-induced oligodendrocyte injury in the CNS. J Neurosci 22:2478–2486. 11923412 10.1523/JNEUROSCI.22-07-02478.2002 PMC6758325

[B60] Leow-Dyke S, Allen C, Denes A, Nilsson O, Maysami S, Bowie AG, Rothwell NJ, Pinteaux E (2012) Neuronal Toll-like receptor 4 signaling induces brain endothelial activation and neutrophil transmigration in vitro. J Neuroinflammation 9:230. 10.1186/1742-2094-9-230 23034047 PMC3481358

[B61] Li Y, Zhang H, Zhang H, Kosturakis AK, Jawad AB, Dougherty PM (2014) Toll-like receptor 4 signaling contributes to Paclitaxel-induced peripheral neuropathy. J Pain 15:712–725. 10.1016/j.jpain.2014.04.001 24755282 PMC4083500

[B62] Li Y, Zhang H, Kosturakis AK, Cassidy RM, Zhang H, Kennamer-Chapman RM, Jawad AB, Colomand CM, Harrison DS, Dougherty PM (2015) MAPK signaling downstream to TLR4 contributes to paclitaxel-induced peripheral neuropathy. Brain Behav Immun 49:255–266. 10.1016/j.bbi.2015.06.003 26065826 PMC4567501

[B63] Lin Y, Lee H, Berg AH, Lisanti MP, Shapiro L, Scherer PE (2000) The lipopolysaccharide-activated toll-like receptor (TLR)-4 induces synthesis of the closely related receptor TLR-2 in adipocytes. J Biol Chem 275:24255–24263. 10.1074/jbc.M002137200 10823826

[B64] Listwak SJ, Rathore P, Herkenham M (2013) Minimal NF-κB activity in neurons. Neuroscience 250:282–299. 10.1016/j.neuroscience.2013.07.013 23872390 PMC3785079

[B65] Liu CY, Mueller MH, Grundy D, Kreis ME (2007) Vagal modulation of intestinal afferent sensitivity to systemic LPS in the rat. Am J Physiol Gastrointest Liver Physiol 292:G1213–G1220. 10.1152/ajpgi.00267.2006 17204546

[B66] Liu S, Gallo DJ, Green AM, Williams DL, Gong X, Shapiro RA, Gambotto AA, Humphris EL, Vodovotz Y, Billiar TR (2002) Role of toll-like receptors in changes in gene expression and NF-kappa B activation in mouse hepatocytes stimulated with lipopolysaccharide. Infect Immun 70:3433–3442. 10.1128/iai.70.7.3433-3442.2002 12065483 PMC128073

[B67] Liu Y, Huang Y, Liu T, Wu H, Cui H, Gautron L (2016) Lipopolysacharide rapidly and completely suppresses AgRP neuron-mediated food intake in male mice. Endocrinology 157:2380–2392. 10.1210/en.2015-2081 27111742 PMC4891783

[B68] Madore C, Joffre C, Delpech JC, De Smedt-Peyrusse V, Aubert A, Coste L, Layé S, Nadjar A (2013) Early morphofunctional plasticity of microglia in response to acute lipopolysaccharide. Brain Behav Immun 34:151–158. 10.1016/j.bbi.2013.08.008 23994463

[B69] Marrone MC, Morabito A, Giustizieri M, Chiurchiù V, Leuti A, Mattioli M, Marinelli S, Riganti L, Lombardi M, Murana E, Totaro A, Piomelli D, Ragozzino D, Oddi S, Maccarrone M, Verderio C, Marinelli S (2017) TRPV1 channels are critical brain inflammation detectors and neuropathic pain biomarkers in mice. Nat Commun 8:15292. 10.1038/ncomms15292 28489079 PMC5436240

[B70] Martin SM, Wilson BC, Chen X, Takahashi Y, Poulin P, Pittman QJ (2000) Vagal CCK and 5-HT(3) receptors are unlikely to mediate LPS or IL-1beta-induced fever. Am J Physiol Regul Integr Comp Physiol 279:R960–R965. 10.1152/ajpregu.2000.279.3.R960 10956254

[B71] Mazzone SB, Undem BJ (2016) Vagal afferent innervation of the airways in health and disease. Physiol Rev 96:975–1024. 10.1152/physrev.00039.2015 27279650 PMC4982036

[B72] Meseguer V, Alpizar YA, Luis E, Tajada S, Denlinger B, Fajardo O, Manenschijn JA, Fernández-Peña C, Talavera A, Kichko T, Navia B, Sánchez A, Señarís R, Reeh P, Pérez-García MT, López-López JR, Voets T, Belmonte C, Talavera K, Viana F (2014) TRPA1 channels mediate acute neurogenic inflammation and pain produced by bacterial endotoxins. Nat Commun 5:3125. 10.1038/ncomms4125 24445575 PMC3905718

[B73] Miyamoto S, Verma IM (1995) Rel/NF-kappa B/I kappa B story. Adv Cancer Res 66:255–292. 7793317

[B74] Muta T, Osaki K, Yamano Y (2002) Translocation t(9;22) (p23;q11) in atypical chronic myeloid leukemia (aCML) presenting osteolytic lesions. Int J Hematol 76:344–348. 10.1007/BF02982694 12463598

[B75] Myers A, Undem B, Kummer W (1996) Anatomical and electrophysiological comparison of the sensory innervation of bronchial and tracheal parasympathetic ganglion neurons. J Auton Nerv Syst 61:162–168. 10.1016/s0165-1838(96)00081-1 8946336

[B76] Ochoa-Cortes F, Ramos-Lomas T, Miranda-Morales M, Spreadbury I, Ibeakanma C, Barajas-Lopez C, Vanner S (2010) Bacterial cell products signal to mouse colonic nociceptive dorsal root ganglia neurons. Am J Physiol Gastrointest Liver Physiol 299:G723–G732. 10.1152/ajpgi.00494.2009 20576919

[B77] Olson JK, Miller SD (2004) Microglia initiate central nervous system innate and adaptive immune responses through multiple TLRs. J Immunol 173:3916–3924. 10.4049/jimmunol.173.6.3916 15356140

[B78] Park BS, Song DH, Kim HM, Choi BS, Lee H, Lee JO (2009) The structural basis of lipopolysaccharide recognition by the TLR4-MD-2 complex. Nature 458:1191–1195. 10.1038/nature07830 19252480

[B79] Poltorak A, He X, Smirnova I, Liu MY, Van Huffel C, Du X, Birdwell D, Alejos E, Silva M, Galanos C, Freudenberg M, Ricciardi-Castagnoli P, Layton B, Beutler B (1998a) Defective LPS signaling in C3H/HeJ and C57BL/10ScCr mice: mutations in Tlr4 gene. Science 282:2085–2088. 10.1126/science.282.5396.2085 9851930

[B80] Poltorak A, Smirnova I, He X, Liu MY, Van Huffel C, McNally O, Birdwell D, Alejos E, Silva M, Du X, Thompson P, Chan EK, Ledesma J, Roe B, Clifton S, Vogel SN, Beutler B (1998b) Genetic and physical mapping of the Lps locus: identification of the toll-4 receptor as a candidate gene in the critical region. Blood Cells Mol Dis 24:340–355. 10.1006/bcmd.1998.0201 10087992

[B81] Poltorak A, Ricciardi-Castagnoli P, Citterio S, Beutler B (2000) Physical contact between lipopolysaccharide and toll-like receptor 4 revealed by genetic complementation. Proc Natl Acad Sci USA 97:2163–2167. 10.1073/pnas.040565397 10681462 PMC15771

[B82] Porter MH, Arnold M, Langhans W (1998a) TNF-alpha tolerance blocks LPS-induced hypophagia but LPS tolerance fails to prevent TNF-alpha-induced hypophagia. Am J Physiol 274:R741–R745. 10.1152/ajpregu.1998.274.3.R741 9530241

[B83] Porter MH, Hrupka BJ, Langhans W, Schwartz GJ (1998b) Vagal and splanchnic afferents are not necessary for the anorexia produced by peripheral IL-1beta, LPS, and MDP. Am J Physiol 275:R384–R389. 10.1152/ajpregu.1998.275.2.R384 9688672

[B84] Prescott SL, Umans BD, Williams EK, Brust RD, Liberles SD (2020) An airway protection program revealed by sweeping genetic control of vagal afferents. Cell 181:574–589.e14. 10.1016/j.cell.2020.03.004 32259485 PMC7197391

[B85] Quan N, Whiteside M, Kim L, Herkenham M (1997) Induction of inhibitory factor kappaBalpha mRNA in the central nervous system after peripheral lipopolysaccharide administration: an in situ hybridization histochemistry study in the rat. Proc Natl Acad Sci USA 94:10985–10990. 10.1073/pnas.94.20.10985 9380746 PMC23556

[B86] Redeker C, Briscoe WH (2019) Interactions between mutant bacterial lipopolysaccharide (LPS-Ra) surface layers: surface vesicles, membrane fusion, and effect of Ca(2+)and temperature. Langmuir 35:15739–15750. 10.1021/acs.langmuir.9b02609 31604373

[B87] Riley TP, Neal-McKinney JM, Buelow DR, Konkel ME, Simasko SM (2013) Capsaicin-sensitive vagal afferent neurons contribute to the detection of pathogenic bacterial colonization in the gut. J Neuroimmunol 257:36–45. 10.1016/j.jneuroim.2013.01.009 23481698 PMC4188534

[B88] Rolls A, Shechter R, London A, Ziv Y, Ronen A, Levy R, Schwartz M (2007) Toll-like receptors modulate adult hippocampal neurogenesis. Nat Cell Biol 9:1081–1088. 10.1038/ncb1629 17704767

[B89] Ross G, Hübschle T, Pehl U, Braun HA, Voigt K, Gerstberger R, Roth J (2003) Fever induction by localized subcutaneous inflammation in guinea pigs: the role of cytokines and prostaglandins. J Appl Physiol (1985) 94:1395–1402. 10.1152/japplphysiol.00485.2002 12482772

[B90] Ruhl CR, Pasko BL, Khan HS, Kindt LM, Stamm CE, Franco LH, Hsia CC, Zhou M, Davis CR, Qin T, Gautron L, Burton MD, Mejia GL, Naik DK, Dussor G, Price TJ, Shiloh MU (2020) Mycobacterium tuberculosis sulfolipid-1 activates nociceptive neurons and induces cough. Cell 181:293–305.e11. 10.1016/j.cell.2020.02.026 32142653 PMC7102531

[B91] Rummel C, Inoue W, Sachot C, Poole S, Hübschle T, Luheshi GN (2008) Selective contribution of interleukin-6 and leptin to brain inflammatory signals induced by systemic LPS injection in mice. J Comp Neurol 511:373–395. 10.1002/cne.21850 18803240

[B92] Sachot C, Poole S, Luheshi GN (2004) Circulating leptin mediates lipopolysaccharide-induced anorexia and fever in rats. J Physiol 561:263–272. 10.1113/jphysiol.2004.074351 15388782 PMC1665347

[B93] Schwartz GJ, Plata-Salamán CR, Langhans W (1997) Subdiaphragmatic vagal deafferentation fails to block feeding-suppressive effects of LPS and IL-1 beta in rats. Am J Physiol 273:R1193–R1198. 10.1152/ajpregu.1997.273.3.R1193 9321903

[B94] Sergeev VG, Akmaev IG (2000) Effects of vagotomy and bacterial lipopolysaccharide on food intake and expression of cyclooxygenase-2 mRNA in rat brain vessels. Bull Exp Biol Med 129:553–555. 10.1007/BF02434874 11022247

[B95] Skelly DT, Hennessy E, Dansereau MA, Cunningham C (2013) A systematic analysis of the peripheral and CNS effects of systemic LPS, IL-1β, [corrected] TNF-α and IL-6 challenges in C57BL/6 mice. PLoS One 8:e69123. 10.1371/journal.pone.0069123 23840908 PMC3698075

[B96] Soldano A, Alpizar YA, Boonen B, Franco L, López-Requena A, Liu G, Mora N, Yaksi E, Voets T, Vennekens R, Hassan BA, Talavera K (2016) Gustatory-mediated avoidance of bacterial lipopolysaccharides via TRPA1 activation in *Drosophila*. Elife 5:e13133. 10.7554/eLife.13133 PMC4907694 27296646

[B97] Stern EL, Quan N, Proescholdt MG, Herkenham M (2000) Spatiotemporal induction patterns of cytokine and related immune signal molecule mRNAs in response to intrastriatal injection of lipopolysaccharide. J Neuroimmunol 106:114–129. 10.1016/s0165-5728(00)00194-6 10814789

[B98] Stirling LC, Forlani G, Baker MD, Wood JN, Matthews EA, Dickenson AH, Nassar MA (2005) Nociceptor-specific gene deletion using heterozygous NaV1.8-Cre recombinase mice. Pain 113:27–36. 10.1016/j.pain.2004.08.015 15621361

[B100] Wang H, Su N, Wang LC, Wu X, Bui S, Nielsen A, Vo HT, Luo Y, Ma XJ (2014) Quantitative ultrasensitive bright-field RNA in situ hybridization with RNAscope. Methods Mol Biol 1211:201–212. 10.1007/978-1-4939-1459-3_16 25218387

[B101] Wang J, Kollarik M, Ru F, Sun H, McNeil B, Dong X, Stephens G, Korolevich S, Brohawn P, Kolbeck R, Undem B (2017) Distinct and common expression of receptors for inflammatory mediators in vagal nodose versus jugular capsaicin-sensitive/TRPV1-positive neurons detected by low input RNA sequencing. PLoS One 12:e0185985. 10.1371/journal.pone.0185985 28982197 PMC5628920

[B102] Wieczorek M, Swiergiel AH, Pournajafi-Nazarloo H, Dunn AJ (2005) Physiological and behavioral responses to interleukin-1beta and LPS in vagotomized mice. Physiol Behav 85:500–511. 10.1016/j.physbeh.2005.05.012 15996692 PMC2293826

[B103] Yao JH, Ye SM, Burgess W, Zachary JF, Kelley KW, Johnson RW (1999) Mice deficient in interleukin-1beta converting enzyme resist anorexia induced by central lipopolysaccharide. Am J Physiol 277:R1435–R1443. 10.1152/ajpregu.1999.277.5.R1435 10564217

[B104] Yin N, Gao Q, Tao W, Chen J, Bi J, Ding F, Wang Z (2020) Paeoniflorin relieves LPS-induced inflammatory pain in mice by inhibiting NLRP3 inflammasome activation via transient receptor potential vanilloid 1. J Leukoc Biol 108:229–241. 10.1002/JLB.3MA0220-355R 32083340

[B105] Yoo KY, Yoo DY, Hwang IK, Park JH, Lee CH, Choi JH, Kwon SH, Her S, Lee YL, Won MH (2011) Time-course alterations of Toll-like receptor 4 and NF-κB p65, and their co-expression in the gerbil hippocampal CA1 region after transient cerebral ischemia. Neurochem Res 36:2417–2426. 10.1007/s11064-011-0569-0 21842272

[B106] Yu S, Undem BJ, Kollarik M (2005) Vagal afferent nerves with nociceptive properties in guinea-pig oesophagus. J Physiol 563:831–842. 10.1113/jphysiol.2004.079574 15649987 PMC1665603

