# Supplementary figures and images for: TLR4 Signaling Selectively and Directly Promotes CGRP Release from Vagal Afferents in the Mouse
Source: eNeuro. 2021 Jan 15;8(1):ENEURO.0254-20.2020. doi: 10.1523/ENEURO.0254-20.2020 (PMC7877464; doi:10.1523/ENEURO.0254-20.2020)

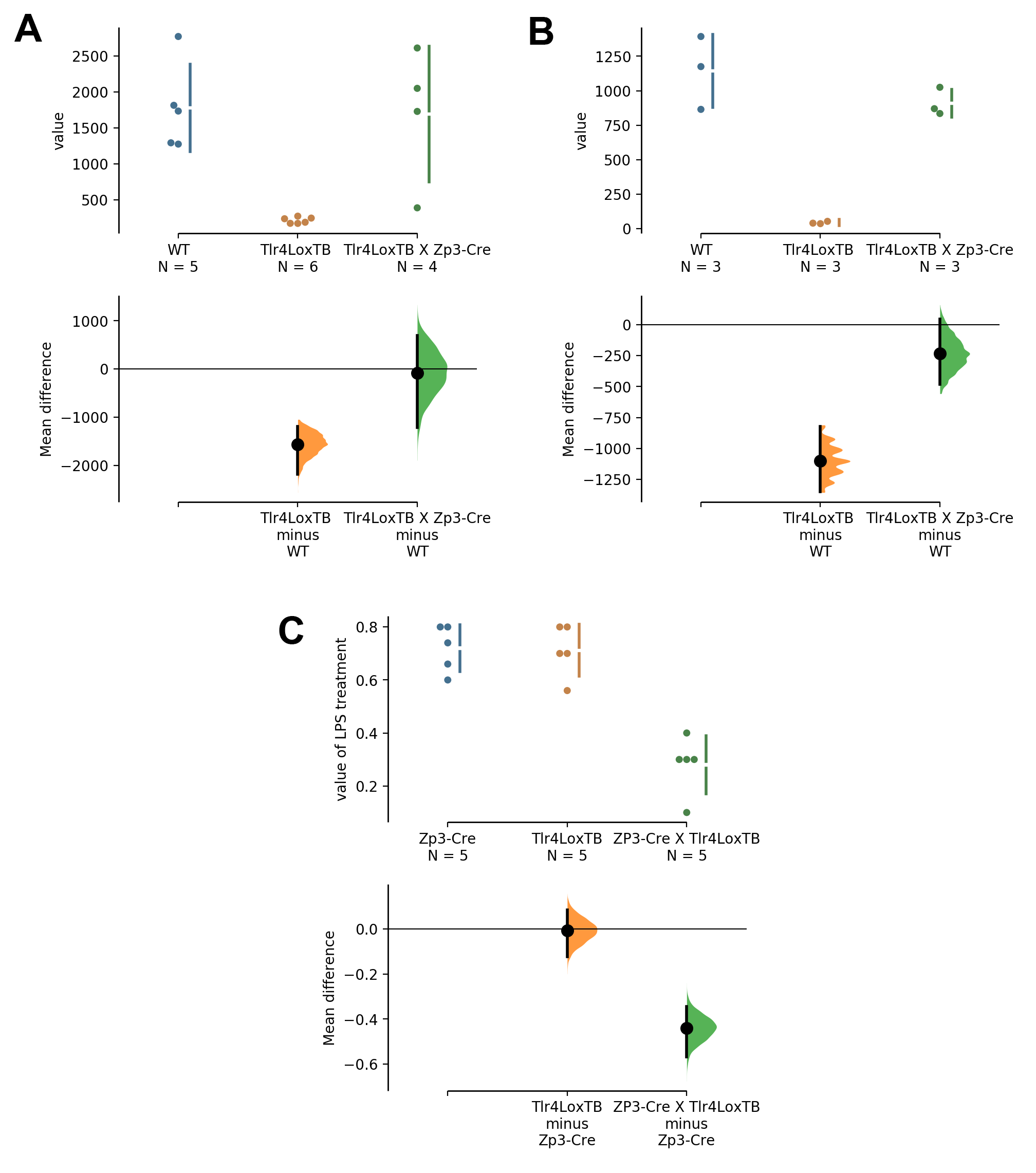

Supplement: Extended Data Figure 1-1 — A, B, Estimation statistic corresponding to Figure 1C,D. Mean difference for two comparisons against the shared control WT are shown in the above Cumming estimation plot. The raw data are plotted on the upper axes. On the lower axes, mean differences are plotted as bootstrap sampling distributions. Each mean difference is depicted as a dot. Each 95% confidence interval is indicated by the ends of the vertical error bars. C, Estimation statistic corresponding to Figure 1E. Mean difference for two comparisons against the shared control Zp3-Cre are shown in the above Cumming estimation plot. The raw data are plotted on the upper axes. On the lower axes, mean differences are plotted as bootstrap sampling distributions. Each mean difference is depicted as a dot. Each 95% confidence interval is indicated by the ends of the vertical error bars. Download Figure 1-1, TIF file. [file enu-eN-NWR-0254-20-s03.tif]

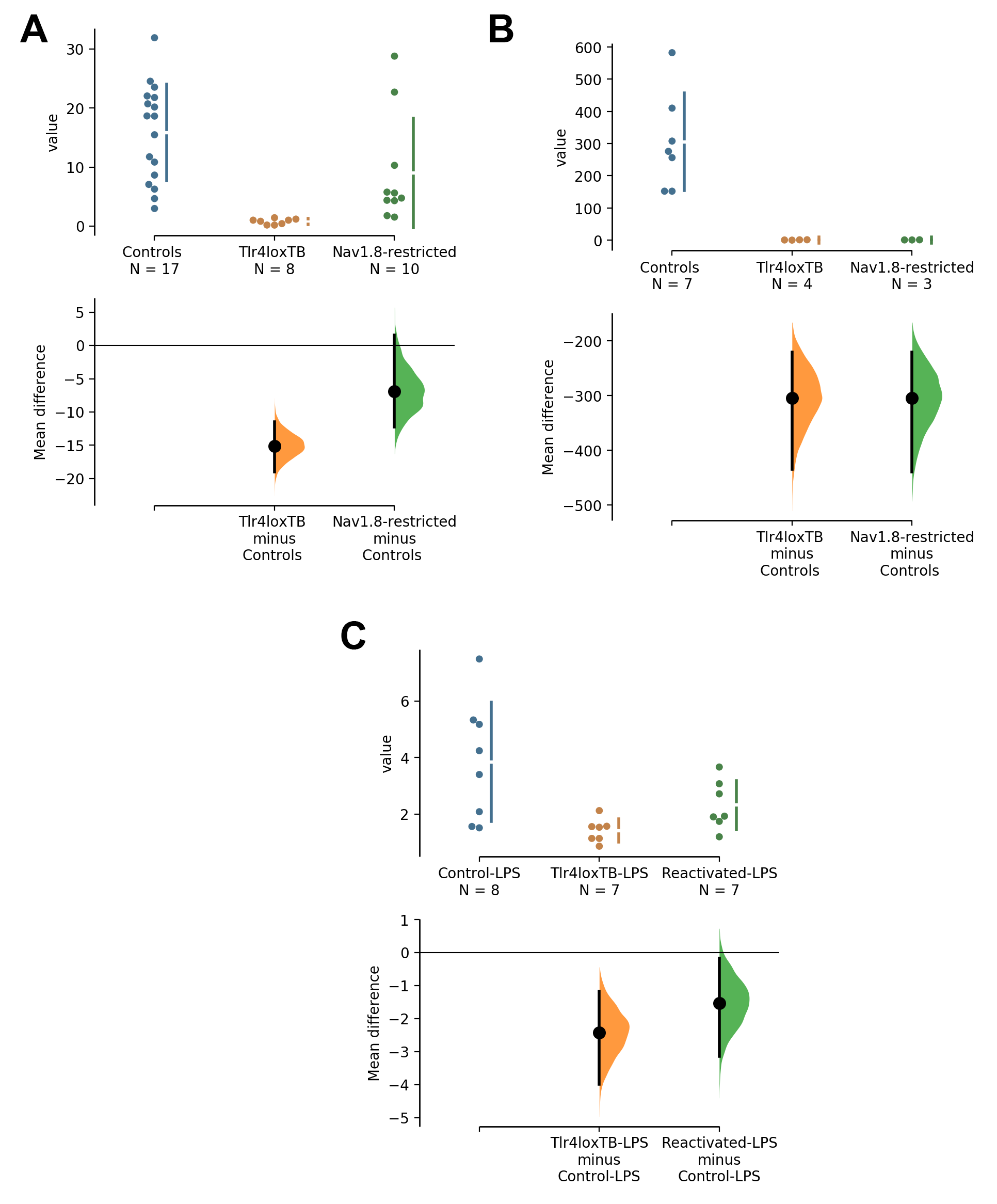

Supplement: Extended Data Figure 2-1 — A, B, Estimation statistic corresponding to Figure 2A,B. Mean difference for two comparisons against the shared controls are shown in the above Cumming estimation plot. The raw data are plotted on the upper axes. On the lower axes, mean differences are plotted as bootstrap sampling distributions. Each mean difference is depicted as a dot. Each 95% confidence interval is indicated by the ends of the vertical error bars. C, Estimation statistic corresponding to Figure 2C. Estimation statistics was calculated in LPS-treated mice. The mean difference for two comparisons against the shared controls are shown in the above Cumming estimation plot. The raw data are plotted on the upper axes. On the lower axes, mean differences are plotted as bootstrap sampling distributions. Each mean difference is depicted as a dot. Each 95% confidence interval is indicated by the ends of the vertical error bars. Download Figure 2-1, TIF file. [file enu-eN-NWR-0254-20-s04.tif]

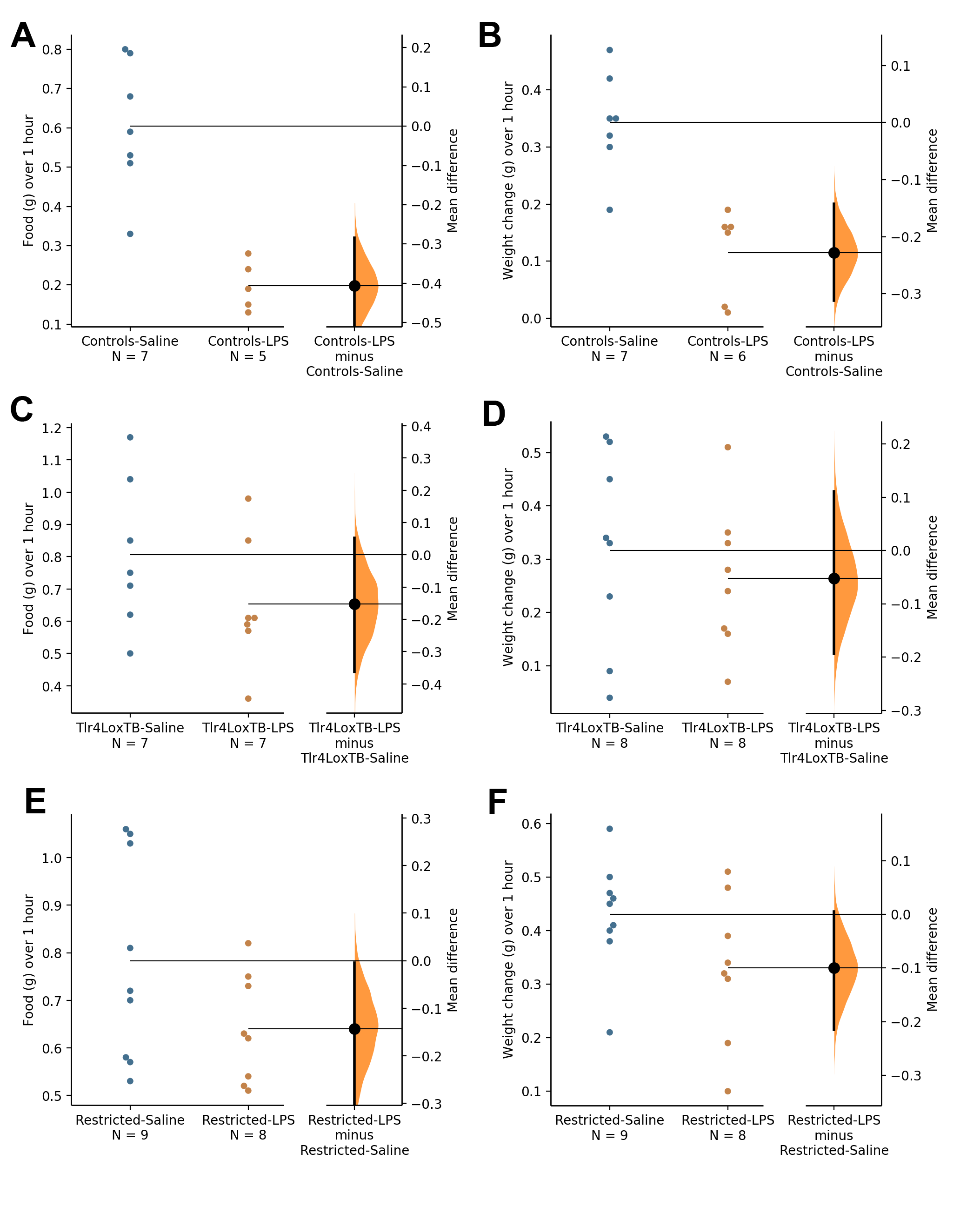

Supplement: Extended Data Figure 3-1 — A, B, Estimation statistic corresponding to Figure 3B,C. The mean difference between controls-saline and controls-LPS for food (A) and weight change (B) are shown in the Gardner–Altman estimation plot. Both groups are plotted on the left axes; the mean difference is plotted on a floating axis on the right as a bootstrap sampling distribution. The mean difference is depicted as a dot; the 95% confidence interval is indicated by the ends of the vertical error bar. C, D, Estimation statistic corresponding to Figure 3E,F. The mean difference between Tlr4LoxTB-Saline and Tlr4LoxTB-LPS for food (A) and weight change (B) are shown in the Gardner–Altman estimation plot. Both groups are plotted on the left axes; the mean difference is plotted on a floating axis on the right as a bootstrap sampling distribution. The mean difference is depicted as a dot; the 95% confidence interval is indicated by the ends of the vertical error bar. E, F, Estimation statistics corresponding to Figure 3H,I. The mean difference between restricted-saline and restricted-LPS for food (E) and weight change (F) are shown in the Gardner–Altman estimation plot. Both groups are plotted on the left axes; the mean difference is plotted on floating axes on the right as a bootstrap sampling distribution. The mean difference is depicted as a dot; the 95% confidence interval is indicated by the ends of the vertical error bar. Download Figure 3-1, TIF file. [file enu-eN-NWR-0254-20-s05.tif]

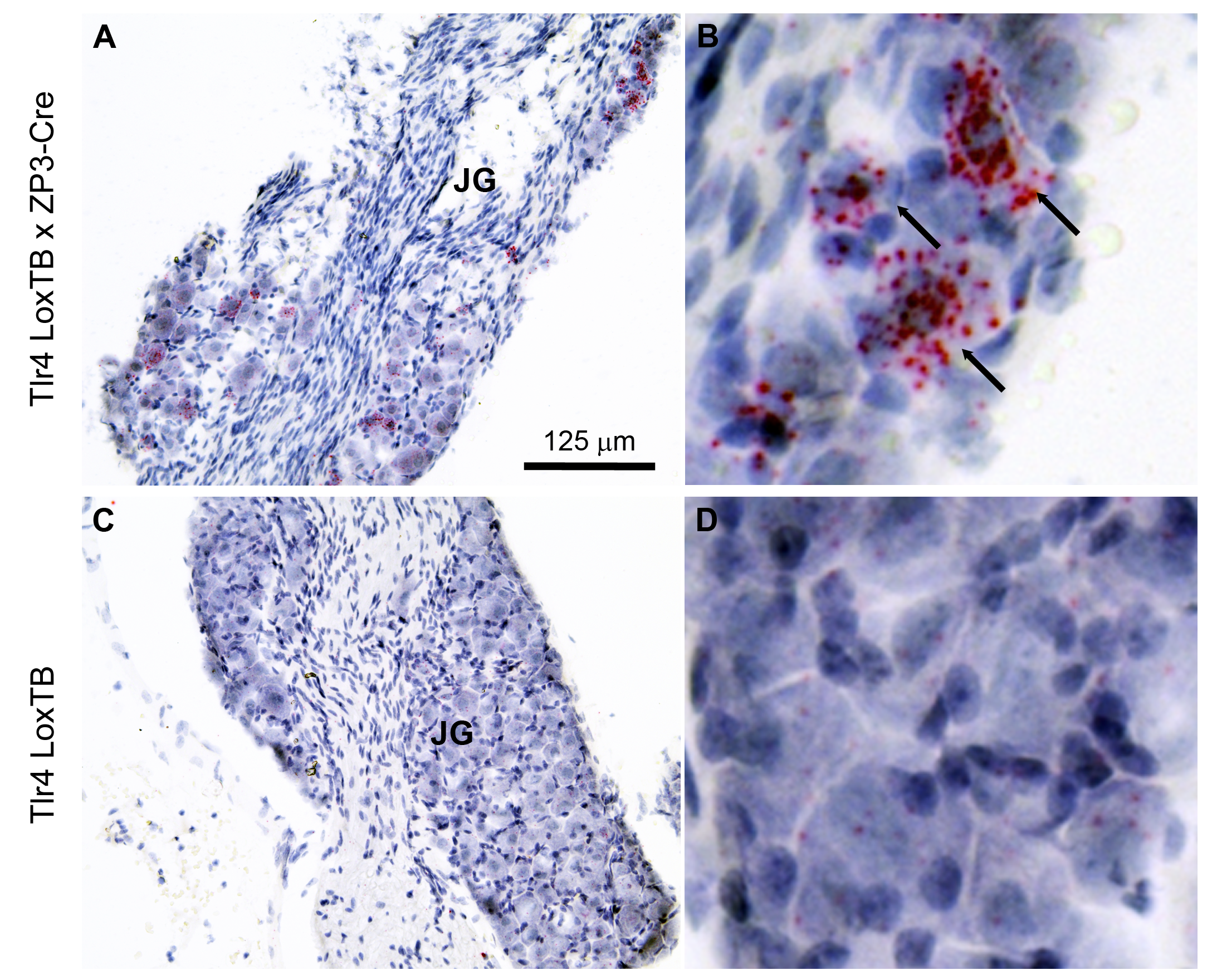

Supplement: Extended Data Figure 4-1 — The specificity of the probe used to detect Tlr4 was validated by comparing by chromogenic ISH for Tlr4 mRNA (red) the ganglia obtained from mice with a globally reactivated allele Tlr4LoxTB (A, B) to that of Tlr4LoxTB mice (C, D). It is evident that robust red precipitates accumulated in Tlr4-positive neuronal profiles indicated by black arrows in reactivated mice. In contrast, the ganglion of Tlr4LoxTB mice was almost entirely devoid of Tlr4 signals. Tissues were counterstained with hematoxylin (purple) and images were collected either under brightfield illumination. The scale bar in A applies to C. JG, jugular ganglion. Download Figure 4-1, TIF file. [file enu-eN-NWR-0254-20-s06.tif]

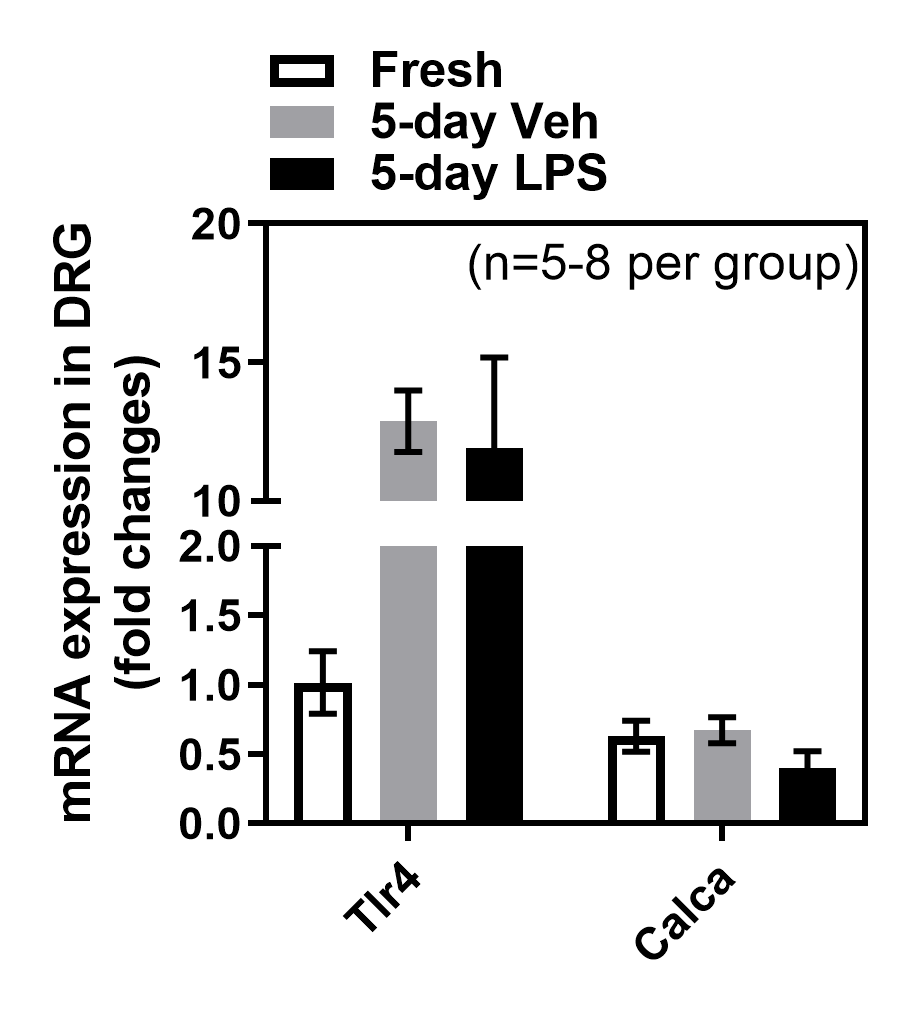

Supplement: Extended Data Figure 7-1 — Q-PCR analysis using Taqman primers (see main text) of dorsal root ganglion (DRG) either freshly collected from WT mice or cultured for 5 d. In all samples, Tlr4 and Calca mRNAs were detected at moderate levels with averaged Ct values of 32. Notably, after 5 d in culture, levels of Tlr4 were robustly stimulated, but Calca remained unchanged. When LPS was applied as described in the main text, Tlr4 and Calca expression levels remained unchanged. Download Figure 7-1, TIF file. [file enu-eN-NWR-0254-20-s07.tif]
